# Modeling the tonotopic map using a two-dimensional array of neural oscillators

**DOI:** 10.3389/fncom.2022.909058

**Published:** 2022-08-24

**Authors:** Dipayan Biswas, V. Srinivasa Chakravarthy, Asit Tarsode

**Affiliations:** ^1^Laboratory for Computational Neuroscience, Department of Biotechnology, Bhupat and Jyoti Mehta School of Biosciences, Indian Institute of Technology Madras, Chennai, India; ^2^Department of Mechanical Engineering, Indian Institute of Technology Madras, Chennai, India

**Keywords:** self-organizing map, tonotopy, modified power coupling, interference, resonance, Hopf oscillator, entrainment, synchronization

## Abstract

We present a model of a tonotopic map known as the Oscillatory Tonotopic Self-Organizing Map (OTSOM). It is a 2-dimensional, self-organizing array of Hopf oscillators, capable of performing a Fourier-like decomposition of the input signal. While the rows in the map encode the input phase, the columns encode frequency. Although Hopf oscillators exhibit resonance to a sinusoidal signal when there is a frequency match, there is no obvious way to also achieve phase tuning. We propose a simple method by which a pair of Hopf oscillators, unilaterally coupled through a coupling scheme termed as modified power coupling, can exhibit tuning to the phase offset of sinusoidal forcing input. The training of OTSOM is performed in 2 stages: while the frequency tuning is adapted in Stage 1, phase tuning is adapted in Stage 2. Earlier tonotopic map models have modeled frequency as an abstract parameter unconnected to any oscillation. By contrast, in OTSOM, frequency tuning emerges as a natural outcome of an underlying resonant process. The OTSOM model can possibly be regarded as an approximation of the tonotopic map found in the primary auditory cortices of mammals, particularly exemplified in the studies of echolocating bats.

## Introduction

The discovery of cortical brain maps in mammalian brains is perhaps one of the first milestones in our understanding of how the brain generates representations of the world. Visual research had discovered a rich hierarchy of maps of various sub-modalities of vision (orientation, curvature, color, and even complex objects) in various visual cortical areas extended over the occipital, parietal, and temporal lobes (Hubel and Wiesel, 1959; Hadjikhani et al., 1998; Wandell et al., 2007; Yue et al., 2020). A similar network of maps of somatotopy was found in the somatosensory areas of the postcentral gyrus and the posterior parietal cortex (Penfield, 1937). These studies have placed on a firm foundation the understanding that sensory information in the brain is often laid out in the form of a system of topographic maps. However, efforts to establish a similar map structure underlying auditory processing—popularly referred to as tonotopic maps—are met with considerable challenges.

Tonotopy begins in the inner ear, in the hair cells laid out along the length of the basilar membrane inside the cochlea (von Bekesy, 1949). Parts of the basilar membrane respond to different frequencies, with the tuning frequency increasing in the apex to the base direction (Ruggero, 1992). Thus, there is a well-established tonotopy in the cochlea, sometimes also referred to as cochleotopy. Beyond the cochlea, there is a hierarchy of areas along the auditory pathway (Clopton et al., 1974; Ehret and Romand, 1996; Palmer and Rees, 2010). Although there is a general agreement that what is mapped in tonotopic maps is the frequencies, other auditory parameters like sound intensity and tuning bandwidth are also explored (Schreiner and Sutter, 1992; Boynton et al., 2015).

Earliest studies on tonotopy focused on frequency tuning, treating it as one of the primary features if not the sole defining feature of auditory response. Merzenich et al. (2018) found a systematic representation of cochlea within the primary auditory cortex of cats. It was observed that frequency bands of the input stimuli are mapped onto rectilinear strips in the auditory cortex. Similar observations were made in the auditory cortex of the gray squirrel (Merzenich et al., 1976). Investigations of the auditory cortex in owl monkeys have discovered a central area with orderly mapping of audible frequencies, circumscribed by areas where neurons show more complex responses than frequency tuning (Imig and Adrian, 1977). However, the exact nature of the complex responses was not elaborated in the last study.

A hierarchically organized network of areas with complex information processing properties was discovered subsequently in the auditory cortex of the bat (Suga, 1990). Contrary to popular belief, bats are not visually blind, although the extent of visual capacity varies with different subspecies of bats. But bats predominantly depend on echolocation to navigate through the spatial world. Bats emit ultrasound pulses in the frequency range of tens of kilohertz, and interpret the spatial world from the echoes returned by the environment. Whereas the delay between the emitted and the received pulse reveals distance, Doppler shift in the echo reveals the relative velocity between the echolocating bat and a target. Pioneering studies of the bat's auditory system by Alvin Novick, James Simmons, Nobuo Suga, and others revealed that these complex auditory functions of the bat are subserved by a well-developed auditory system (Novick and Vaisnys, 1964; Suga, 1990; Suga et al., 1997; Bates et al., 2011; Simmons, 2012).

Studies by Nobuo Suga and colleagues with the mustached bat described an elaborate network of auditory cortical areas with an intrinsic hierarchy not very different from that of the primate visual system (Hubel and Wiesel, 1959). The mustached bat emits composite pulses that have an initial Constant Frequency (CF) section terminated by a Frequency Modulated (FM) section. There is a cortical region in which neurons respond only to certain combinations of frequencies and amplitudes of echoes. There is a region where neurons respond only to frequency differences between the emitted pulse and its echoes, probably encoding Doppler shift. In another region, there are neurons that respond to the time delay between the emitted pulse and the echo, perhaps encoding the distance to the target. The gains obtained from the study of the bat's auditory system are not yet fully exploited in unraveling the auditory architecture of the brains of higher mammals and humans.

In the domain of computational modeling, one of the earliest tonotopic map models used a Self-Organizing Map (SOM) model to model the auditory cortex of mustached bats (Ritter et al., 1992). The model adopted a simplistic view of the organization of the bat's auditory cortex—that the input frequencies are mapped along a rectilinear strip of the cortex—and shows how such a mapping can be realized using a rectangular SOM model. A key limitation of the model is the representation of frequency as an explicit scalar variable and not as an implicit temporal property of an ongoing oscillation. The SOM approach to modeling tonotopy was extended to construct a model of a “phonetic typewriter” (Kohonen, 1988). Palakal et al. (1995) presented a tonotopic map model also based on the SOM approach, describing neural tuning to both frequency and delay. Here, too, frequency and delay are explicitly represented as scalar variables, and not as implicit temporal properties of a signal.

Models tend to make simplifying assumptions of the processes they aim to model, but it is rather unnatural to model frequency as simply a number without explicitly modeling the oscillation that the frequency refers to. A tonotopic map is primarily a response to tones, which are oscillations. Oscillatory activities are found at all levels in the auditory pathway, from cochlea to inferior colliculus to higher auditory cortical areas. Essentially, the active nonlinearity exhibited by the outer hair cells of cochlea is well recognized to be modeled using Hopf oscillators critically poised at the bifurcation regime (Eguíluz et al., 2000; Frank et al., 2001; Kern and Stoop, 2003; Lerud et al., 2019). A network of coupled ‘neural oscillators' can be reduced to a canonical model when it operates near a multiple Andronov-Hopf bifurcation point (Aronson et al., 1990; Hoppensteadt and Izhikevich, 1996). The neural oscillators are generally representative of distinguishably interconnected population excitatory and inhibitory neurons (Hoppensteadt and Izhikevich, 1997). Gradient frequency neural networks (GrFNN) are an attempt to model auditory signal processing using a network of such a heterogeneous frequency canonical model of oscillators (Large et al., 2010; Kim and Large, 2015, 2019, 2021; Farokhniaee et al., 2020). However, these are single-unit models of oscillation and not map models.

From the aforementioned quick review of auditory response models, we understand that there are tonotopic map models that do not explicitly model the underlying oscillation, and there are oscillatory models at a single-unit level that is not extended to map models. Thus, the challenge of constructing a tonotopic map model of nonlinear oscillators is still unrealized, which becomes the motivation of this work.

We present a tonotopic map model consisting of a 2-dimensional array of nonlinear oscillators. Specifically, we choose the Hopf oscillators since these oscillators have been extensively used to model auditory responses (Frank et al., 2001; Large et al., 2010; Fredrickson-hemsing et al., 2012; Kim and Large, 2015; Farokhniaee et al., 2020). In the following methods section, we have presented the dynamics of the OTSOM model along with the dynamical analysis of a single unit of the OTSOM model and the modified power coupling strategy along with the modified Hebbian learning rule to train it. The dynamical analysis of the two stages to train the characterizing frequencies and the phases of the model is presented thereafter. The numerical analysis from the unit level to the network level is presented in the subsequent results section.

## Methods

The conventional self-organizing maps are known for their special characteristic of organizing internally represented features on a spatial scale, i.e., the neurons representing similar abstract features in the input data organize themselves spatially close to each other through competitive learning (Kohonen, 1998). Typically, SOM models perform dimensionality reduction of a high dimensional input vector by projecting it onto a low dimensional spatial map space. In other words, the input data points located nearby in the *N*-dimensional Euclidean space get mapped onto nearby neurons in the map. The map space can be maximum up to 3 dimensional as it is not easily visualized beyond 3 dimensions. Also, brain maps are typically 2-dimensional, referring typically to cortical sheets of neurons, or mildly 3-dimensional, if the small cortical thickness is included.

The objective of our modeling study is to propose a dynamical self-organizing map model, which can organize the features of complex sinusoidal signals on a 2-dimensional grid of nonlinear oscillators. Signals of any duration have a static representation in the Fourier space or frequency domain. The proposed model is capable of organizing features of complex sinusoidal signals, such as frequency and phase offset, in terms of the parameters of intrinsic dynamics of the single neural oscillator and the connectivity parameters during the training phase. During testing, the trained map model can represent the features of any composite signal with multiple frequency components.

### Oscillatory tonotopic self-organizing map (OTSOM)

**Figure 1:**
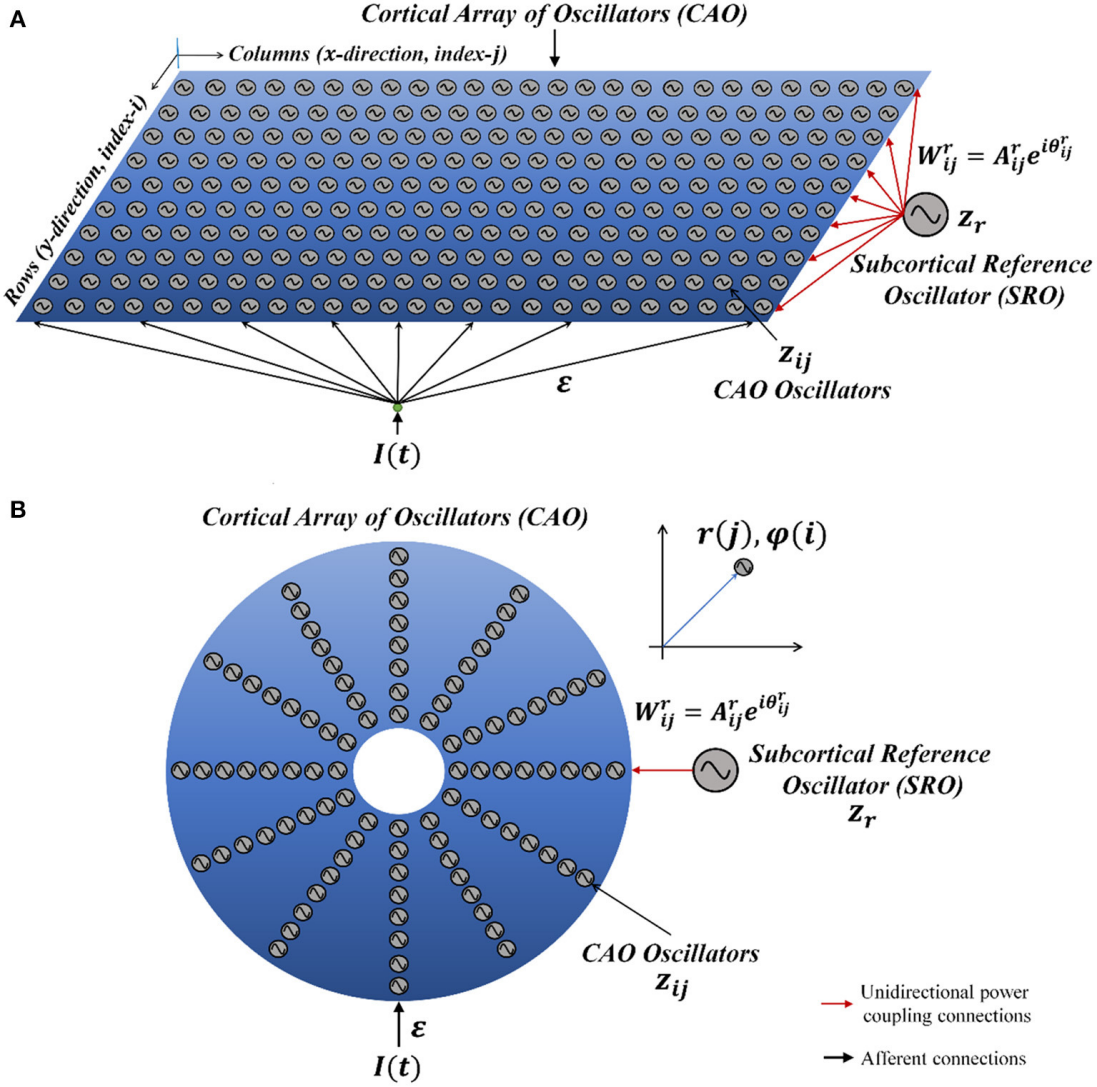
The network architecture of Oscillatory Tonotopic Self-Organizing Map (OTSOM). The model principally contains two types of oscillators: an array of cortical oscillators (CAO) and a Subcortical Reference Oscillator (SRO). The SRO projects unilateral, power-coupling connections to the CAO oscillators. The external input perturbs the oscillators in CAO through uniform afferent connections. The CAO oscillators can be visualized to be organized either in a 2D array **(A)** or in a concentric circular array **(B)**. The radial and the angular position of a CAO oscillator in the concentric circular array is the same as its position along x and y axis, respectively, in the 2D array organization of the cortical array.

The Oscillatory Tonotopic Self-Organizing Map (OTSOM) model consists of a 2D array of Hopf oscillators (Strogatz, 1994) - the “Cortical Array of Oscillators” (CAO) - operating at supercritical Hopf regime as described in Figure 1. A single isolated oscillator located apart from the CAO is interpreted as a subcortical oscillator, and labeled as Subcortical Reference Oscillator (SRO) since it serves as a reference for the phases of the cortical oscillators. Although there are no lateral interactions between the oscillators in CAO, the SRO projects unilateral, trainable connections to all the oscillators in CAO. Thus, each oscillator in CAO receives two inputs: (1) from the SRO (termed as *I*_*r*_) and (2) from the external input (termed as *I*_*e*_) (Figure 1). Neurobiologically, *I*_*e*_ and *I*_*r*_ represent the afferent neuronal signal, conveying auditory stimulus and the thalamic projection to the auditory cortex, respectively. The connections from the external input to the CAO oscillators have uniform fixed weights.

Before going into more details of the dynamics of the OTSOM model, let us first understand the dynamics of the constituting components of the OTSOM model. As mentioned before, the CAO oscillators and the SRO are Hopf oscillators. A single Hopf oscillator can be represented in Cartesian (Equations 1a, b) and polar coordinates (Equations 2a, b), respectively, as follows:


(1a) $$\begin{array}{l} \dot{x}=\left(\mu \;-\;\beta_{1}\left(x^{2}+y^{2}\right)\right)x-\omega y \end{array}$$

(1b) $$\begin{array}{l} \dot{y}=\left(\mu -\beta_{1}\left(x^{2}+y^{2}\right)\right)y+\omega x \end{array}$$

(2a) $$\begin{array}{l} \dot{r}=\left(\mu \;-\;\beta_{1}r^{2}\right)r \end{array}$$

(2b) $$\begin{array}{l} \dot{\emptyset }=\omega \end{array}$$

where (*x, y*) are cartesian coordinate variables and (*r*, ∅) are polar coordinate variables; ω defines the angular velocity or the natural frequency of the oscillator. Refer to the Supplementary Table S1 for the variable/parameter notations and representations. The parameters μ and β_1_ determine the dynamic regime of the Hopf oscillator: for μ = 0, β_1_ > 0, it operates in critical Hopf regime; for μ > 0, β_1_ > 0, it operates in supercritical Hopf regime, and, when μ = 0, β_1_ = 0, it is a simple harmonic oscillator (Kim and Large, 2015).

Combining x and y of Equation (1) into a complex number, *z* = *x* + *iy*, Hopf oscillator dynamics can simply be represented on a complex plane elegantly as:


(3) $$\begin{array}{l} \dot{z}\;=\;\left(\mu \;-\;\beta_{1}|z{|}^{2}\;+\;i\omega \right)z \end{array}$$

where $i=\sqrt{-1}$. A pair of coupled Hopf oscillators can principally exhibit two types of dynamical phenomena: synchronization and entrainment.

A generalized definition of synchronization has been introduced in our previous study (Biswas et al., 2021), which states that any two oscillators can be claimed to be synchronized irrespective of their intrinsic oscillation frequencies if they maintain any of the following phase relationships constant, ∅_1_ − ∅_2_, *m*∅_1_ − *n*∅_2_, $\frac{\emptyset_{1}}{\omega_{1}}-\frac{\emptyset_{2}}{\omega_{2}}$; *m* and *n* are natural numbers. Whereas entrainment is the dynamical characteristic of an oscillator, while the frequency of oscillation of the oscillator gradually changes from its natural frequency of oscillation to a new value when the oscillator is either coupled with another oscillator or perturbed by an external oscillatory input of a different frequency. Real valued symmetric coupling yields in phase (0°) oscillation for positive coupling, and out of phase (180°) oscillation for negative coupling, between two isochronous oscillators, whereas the same pair can phase-lock at any arbitrary phase difference if coupled through ‘complex coupling' strategy (Biswas et al., 2021).

To produce phase-locked dynamics from a pair of oscillators with unequal natural frequencies requires a special kind of complex coupling strategy labeled as ‘power coupling' (Biswas et al., 2021). A pair of oscillators coupled through power coupling is defined as:


(4a) $$\begin{array}{l} \dot{z_{1}}\;=\;\left(\mu \;-\;\beta_{1}|z_{1}{|}^{2}\;+\;i\omega_{1}\right)z_{1}\;+\;W_{12}{z_{2}}^{\frac{\omega_{1}}{\omega_{2}}} \end{array}$$

(4b) $$\begin{array}{l} \dot{z_{2}}\;=\;\left(\mu -\;\beta_{1}|z_{2}{|}^{2}\;+\;i\omega_{2}\right)z_{2}\;+\;W_{21}{z_{1}}^{\frac{\omega_{2}}{\omega_{1}}} \end{array}$$

where, $W_{12}=A_{12}e^{i\frac{\theta_{12}}{\omega_{2}}}$, $W_{21}=A_{21}e^{i\frac{\theta_{21}}{\omega_{1}}}$ represent the complex power coupling coefficients on the feedforward and feedback branches. Considering θ_12_ = −θ_21_, and *A*_12_ = *A*_21_, it has been shown that the pair of oscillators can phase-lock at any of the solutions of the equation: $\frac{\dot{\emptyset_{1}}}{\omega_{1}}-\frac{\dot{\emptyset_{2}}}{\omega_{2}}=0,$ depending on the initial condition. The analytic solution of this problem gets increasingly complicated as the number of oscillators in the network increases. Another limitation of the original power-coupling strategy is that it does not ensure synchronization, while the coupled oscillators are entrained to a new frequency of oscillation.

**Figure 2:**
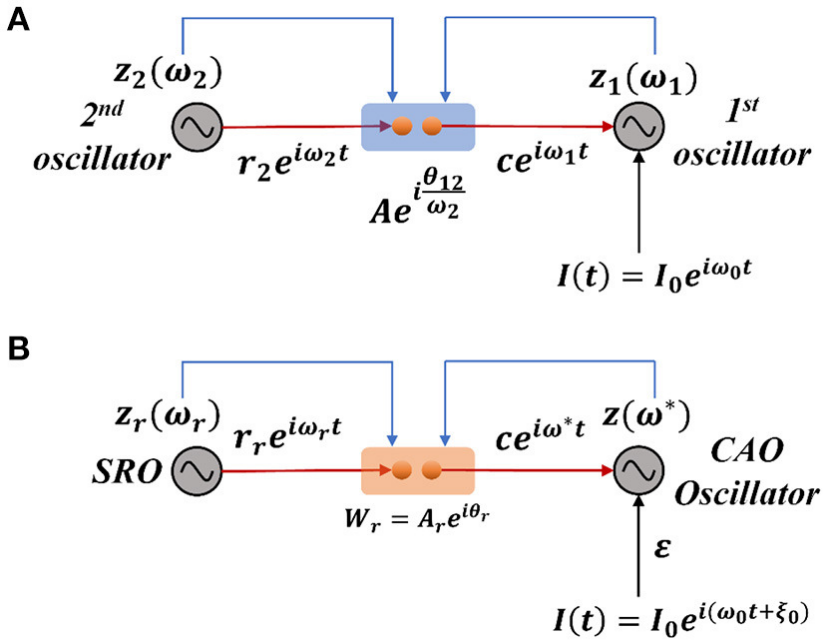
**(A)** The schematic diagram of a pair of Hopf oscillators, unilaterally coupled through conventional power coupling strategy. Here, *c* is typically $A{\left(\frac{\mu }{\beta_{1}}\right)}^{\frac{\omega_{1}}{2\omega_{2}}}$ at a steady state. **(B)** The fundamental building block of the OTSOM model. The single CAO oscillator receives inputs from two sources: one from the SRO and the other from the external input signal. The two differences between these two frameworks are the coupling coefficients; the natural frequency of the SRO is eliminated from the denominator of the angle of the coupling coefficient, and the second being the actual frequency of oscillation of the CAO oscillator is used to readjust the complex activation of the SRO (Subplot A) instead of the natural frequency of the post-synaptic oscillator (Subplot B). The neurobiological interpretation of the proposed model architecture can be brought about by comparing the SRO with the neural oscillator of the thalamic nuclei, which generally have long-range divergent projections to cortical columns, while the CAO is compared with the 2-dimensional array of cortical columns in the auditory cortex.

Considering a simplified scenario of a pair of Hopf oscillators coupled through weak unilateral power coupling, the one receiving input from the other oscillator through power coupling is being perturbed by a strong complex sinusoidal external input signal with frequency close to the natural frequency of the oscillator as depicted in Figure 2A.

We will now try to show the pair of oscillators with an external sinusoidal input as shown in Figure 2A poses a new difficulty in phase-locking not faced by a pair of oscillators with power coupling (Equations 4a, b). Consider the situation in Figure 2A, where the 2nd oscillator sends a unilateral power coupling connection to the 1st oscillator, which, in addition, receives a complex sinusoidal signal of frequency ω_0_ as external input. Note that, although the output of the 2nd oscillator has the frequency ω_2_, after the power coupling connection, the signal frequency changes to ω_1_. Thus, the 1st oscillator receives two sinusoidal inputs – of frequencies ω_0_ and ω_1_. Therefore, the 1st oscillator does not simply entrain to the external input due to the interference from the 2nd oscillator. In order to fix this problem, it turns out that we need a more general power coupling rule than the original one.

### Modified power coupling

To make the original power-coupling rule (Biswas et al., 2021) more generalized, and to ensure synchronization in a pair of power-coupled oscillators even after entrainment, a modified version of the power-coupling strategy is proposed. For a pair of bilaterally coupled Hopf oscillators, the modified power-coupling mechanism is given as:


(5a) $$\begin{array}{l} \dot{z_{1}}\;=\;\left(\mu \;-\;\beta_{1}|z_{1}{|}^{2}\;+\;i\omega_{1}\right)z_{1}\;+\;W_{12}{z_{2}}^{\frac{\omega_{1}^{*}}{\omega_{2}^{*}}} \end{array}$$

(5b) $$\begin{array}{l} \dot{z_{2}}\;=\;\left(\mu \;-\;\beta_{1}|z_{2}{|}^{2}\;+\;i\omega_{2}\right)z_{2}\;+\;W_{21}{z_{1}}^{\frac{\omega_{1}^{*}}{\omega_{2}^{*}}} \end{array}$$

where $W_{12}=A_{12}e^{i\frac{\theta_{12}}{\omega_{2}^{*}}}$, $W_{21}=A_{21}e^{i\frac{\theta_{21}}{\omega_{1}^{*}}}$, the only modification being $\omega_{1}^{*}$ and $\omega_{2}^{*}$ are actual frequencies of oscillation instead of natural frequencies. A new set of dynamic equations (Equations 8a, b) defines $\omega_{1}^{*}$ and $\omega_{2}^{*}$. The overall dynamics of a pair of Hopf oscillators coupled through modified power-coupling is represented in polar coordinates as follows.


(6a) $$\begin{array}{l} \dot{r_{1}}\;=\;\left(\mu -\;\beta_{1}{r_{1}}^{2}\right)r_{1}\;+\;A_{12}{r_{2}}^{\frac{\omega_{1}^{*}}{\omega_{2}^{*}}}\cos\omega_{1}^{*}\left(\frac{\theta_{12}}{\omega_{1}^{*}\omega_{2}^{*}}\;+\;\frac{\emptyset_{2}}{\omega_{2}^{*}}-\frac{\emptyset_{1}}{\omega_{1}^{*}}\right) \end{array}$$

(6b) $$\begin{array}{l} \dot{\emptyset_{1}}\;=\;\omega_{1}\;+\;A_{12}\frac{{r_{2}}^{\frac{\omega_{1}^{*}}{\omega_{2}^{*}}}}{r_{1}}\sin\omega_{1}^{*}\left(\frac{\theta_{12}}{\omega_{1}^{*}\omega_{2}^{*}}\;+\;\frac{\emptyset_{2}}{\omega_{2}^{*}}\;-\;\frac{\emptyset_{1}}{\omega_{1}^{*}}\right) \end{array}$$

(7a) $$\begin{array}{l} \dot{r_{2}}\;=\;\left(\mu \;-\beta_{1}{r_{2}}^{2}\right)r_{2}\;+\;A_{21}{r_{1}}^{\frac{\omega_{2}^{*}}{\omega_{1}^{*}}}\cos\omega_{2}^{*}\left(\frac{\theta_{21}}{\omega_{1}^{*}\omega_{2}^{*}}\;+\;\frac{\emptyset_{1}}{\omega_{1}^{*}}-\frac{\emptyset_{2}}{\omega_{2}^{*}}\right) \end{array}$$

(7b) $$\begin{array}{l} \dot{\emptyset_{2}}\;=\;\omega_{2}\;+\;A_{21}\frac{{r_{1}}^{\frac{\omega_{2}^{*}}{\omega_{1}^{*}}}}{r_{2}}\sin\omega_{2}^{*}\left(\frac{\theta_{21}}{\omega_{1}^{*}\omega_{2}^{*}}\;+\;\frac{\emptyset_{1}}{\omega_{1}^{*}}\;-\;\frac{\emptyset_{2}}{\omega_{2}^{*}}\right) \end{array}$$

(8a) $$\begin{array}{l} \tau_{\omega }\dot{\omega_{1}^{*}}\;=\;-\;\omega_{1}^{*}\;+\;\omega_{1}\;+\;A_{12}\frac{{r_{2}}^{\frac{\omega_{1}^{*}}{\omega_{2}^{*}}}}{r_{1}}\sin\omega_{1}^{*}\left(\frac{\theta_{12}}{\omega_{1}^{*}\omega_{2}^{*}}\;+\;\frac{\emptyset_{2}}{\omega_{2}^{*}}\;-\;\frac{\emptyset_{1}}{\omega_{1}^{*}}\right) \end{array}$$

(8b) $$\begin{array}{l} \tau_{\omega }\dot{\omega_{2}^{*}}\;=\;-\;\omega_{2}^{*}\;+\;\omega_{2}\;+\;A_{21}\frac{{r_{1}}^{\frac{\omega_{2}^{*}}{\omega_{1}^{*}}}}{r_{2}}\sin\omega_{2}^{*}\left(\frac{\theta_{21}}{\omega_{1}^{*}\omega_{2}^{*}}\;+\;\frac{\emptyset_{1}}{\omega_{1}^{*}}\;-\;\frac{\emptyset_{2}}{\omega_{2}^{*}}\right) \end{array}$$

where τ_ω_ is the time constant for $\omega_{1}^{*}$ and $\omega_{2}^{*}$. It is evident that, without Equations 8a and 8b, the modified power-coupling is functionally the same as the conventional one as ω^*^ remains ω. It can also be observed that neither of the oscillators can get entrained to a new frequency of oscillation, which makes a pair of bilaterally coupled Hopf oscillators through modified power coupling functionally the same as a pair of bilaterally coupled Hopf oscillators through conventional power coupling (refer to Appendix 1).

In the subsequent sections, the details of the intrinsic dynamics and the training framework of the OSTOM model are described. The dynamics of a typical CAO oscillator is given as:


(9) $$\begin{array}{l} \dot{z_{ij}}\;=\;\left(\mu \;-\beta_{1}|z_{ij}{|}^{2}\right)z_{ij}\;+\;i\omega_{ij}z_{ij}\;+\;W_{ij}^{r}{z_{r}}^{\frac{\omega_{ij}^{*}}{\omega_{r}}}\;+\;\varepsilon I\left(t\right) \end{array}$$

The first two terms on the RHS denote the intrinsic dynamics of the Hopf oscillator, the third term represents the input from SRO ($I_{ij}^{r}=W_{ij}^{r}{z_{r}}^{\frac{\omega_{ij}^{*}}{\omega_{r}}}$), the fourth term represents the aggregate external input (*I*_*e*_ = ε*I*(*t*)), where *I*(*t*) is the actual external input. Note that only the SRO input is given *via* modified power coupling, whereas the external input *I*(*t*) is presented directly with a multiplicative factor, ε.

Similarly, the dynamics of the SRO is given as:


(10) $$\begin{array}{l} \dot{z_{r}}\;=\;\left(\mu_{r}\;-\;\beta_{1r}|z_{r}{|}^{2}\right)z_{r}\;+\;i\omega_{r}z_{r} \end{array}$$

The activation of the CAO oscillator at location (*i, j*) is defined as $z_{ij}=x_{ij}+iy_{ij}=r_{ij}e^{i\emptyset_{ij}}$; similarly, the activation of the SRO oscillator is $z_{r}=x_{r}+iy_{r}=r_{r}e^{i\emptyset_{r}}$. Intrinsic dynamics of the CAO oscillator is defined by the parameters μ, β_1_, and ω_*ij*_. Note that μ and β_1_ are the same for all CAO oscillators, but ω_*ij*_ is different. The intrinsic dynamics of the SRO oscillator is defined by μ_*r*_, β_1*r*_, and ω_*r*_ parameters. $W_{ij}^{r}$ is the complex power-coupling weight from the reference oscillator to the oscillator at (*i, j*) in CAO, where $W_{ij}^{r}=A_{ij}^{r}e^{i\theta_{ij}^{r}}$.

The reason behind dropping the actual frequency of the presynaptic oscillator from the denominator of the angle of the complex coupling coefficient will be justified in the following sections.

The Cartesian and the polar coordinate representations are respectively.


(9a) $$\begin{array}{l} \dot{x_{ij}}\;=\;\left(\mu -\beta_{1}\left(x_{ij}^{2}\;+\;y_{ij}^{2}\right)\right)x_{ij}\;-\;\omega_{ij}y_{ij}\\ +A_{ij}^{r}{\left(x_{r}^{2}\;+\;y_{r}^{2}\right)}^{\frac{\omega_{ij}^{*}}{2\omega_{r}}}\cos\left(\theta_{ij}^{r}\;+\;\frac{\omega_{ij}^{*}}{\omega_{r}}\frac{{\text{y}}_{\text{r}}^{2}}{{\text{x}}_{\text{r}}^{2}}\right)\;+\;\varepsilon \;imag\left(I\left(t\right)\right) \end{array}$$

(9b) $$\begin{array}{l} \dot{y_{ij}}\;=\;\left(\mu -\beta_{1}\left(x_{ij}^{2}\;+\;y_{ij}^{2}\right)\right)y_{ij}\;+\;\omega_{ij}x_{ij}\\ +A_{ij}^{r}{\left(x_{r}^{2}\;+\;y_{r}^{2}\right)}^{\frac{\omega_{ij}^{*}}{2\omega_{r}}}\sin\left(\theta_{ij}^{r}\;+\;\frac{\omega_{ij}^{*}}{\omega_{r}}\frac{{\text{y}}_{\text{r}}^{2}}{{\text{x}}_{\text{r}}^{2}}\right)\;+\;\varepsilon \;real\left(I\left(t\right)\right) \end{array}$$

(9c) $$\begin{array}{l} \dot{r_{ij}}\;=\;\left(\mu -\beta_{1}{r_{ij}}^{2}\right)r_{ij}\;+\;A_{ij}^{r}{r_{r}}^{\frac{\omega_{ij}^{*}}{\omega_{r}}}\cos\omega_{ij}^{*}\left(\frac{\theta_{ij}^{r}}{\omega_{ij}^{*}}\;+\;\frac{\emptyset_{r}}{\omega_{r}}\;-\;\frac{\emptyset_{ij}}{\omega_{ij}^{*}}\right)\\ +\varepsilon real\left(I\left(t\right)e^{-i\emptyset_{ij}}\right) \end{array}$$

(9d) $$\begin{array}{l} \dot{\emptyset_{ij}}\;=\;\omega_{ij}\;+\;A_{ij}^{r}\frac{{r_{r}}^{\frac{\omega_{ij}^{*}}{\omega_{r}}}}{r_{ij}}\sin\omega_{ij}^{*}\left(\frac{\theta_{ij}^{r}}{\omega_{ij}^{*}}\;+\;\frac{\emptyset_{r}}{\omega_{r}}\;-\;\frac{\emptyset_{ij}}{\omega_{ij}^{*}}\right)\\ +\;\frac{\varepsilon }{r_{ij}}imag\left(I\left(t\right)e^{-i\emptyset_{ij}}\right) \end{array}$$

(10a) $$\begin{array}{l} \dot{r_{r}}\;=\;\left(\mu_{r}\;-\;\beta_{1r}{r_{r}}^{2}\right)r_{r} \end{array}$$

(10b) $$\begin{array}{l} \dot{\emptyset_{r}}\;=\;\omega_{r} \end{array}$$

(11) $$\begin{array}{l} \tau_{\omega }\dot{\omega_{ij}^{*}}=\;-\;\omega_{ij}^{*}\;+\;\omega_{ij}\;+\;A_{ij}^{r}\frac{{r_{r}}^{\frac{\omega_{ij}^{*}}{\omega_{r}}}}{r_{ij}}\sin\omega_{ij}^{*}\left(\frac{\theta_{ij}^{r}}{\omega_{ij}^{*}}\;+\;\frac{\emptyset_{r}}{\omega_{r}}\;-\;\frac{\emptyset_{ij}}{\omega_{ij}^{*}}\right)\\ +\frac{\varepsilon }{r_{ij}}imag\left(I\left(t\right)e^{-i\emptyset_{ij}}\right) \end{array}$$

The uniform, real-valued, afferent connections from the external input (*I*(*t*)) to the CAO oscillators are ε. $\omega_{ij}^{*}$ and $\omega_{r}^{*}$ are the actual frequencies of the CAO oscillators and the SRO, respectively. As $\omega_{r}^{*}$ remains ω_*r*_, it is replaced with ω_*r*_. The actual frequency of oscillation of the CAO oscillator can be entrained to the frequency of the external perturbation. This entrainment property of the Hopf oscillator is utilized to realize the framework of the proposed model, which will be discussed in detail in the later sections. Before elaborating the details of the training framework of the OTSOM model, we are going to briefly analyze the intrinsic dynamical properties of the single unit (the SRO unilaterally coupled to a CAO oscillator) of the model.

### Dynamical response of a single unit

The single unit, which is the fundamental building block of the whole OSTOM model, is constituted of a single oscillator in the CAO, which receives two inputs: (1) from the SRO *via* modified power coupling and (2) from the external input. The SRO is coupled to a CAO oscillator unilaterally through a modified power coupling connection (Equations 9–11). The output of the SRO, after passing through modified power-coupling connection, takes on the same frequency as that of the CAO oscillator it projects to. The CAO oscillator is also driven by an external input signal through a real-valued afferent connection (Figure 2B). The external input is a complex sinusoidal signal.

We now investigate the steady state response of the single unit by analyzing (Equations 12–14). Let the external input signal $I\left(t\right)=I_{0}e^{i\emptyset_{0}}=I_{0}e^{i\left(\omega_{0}t+\xi_{0}\right)}$ where ω_0_ and ξ_0_ are, respectively, the frequency and the phase offset of the complex sinusoidal signal. Introducing the following variables,

- the relative phase of the CAO oscillator w.r.t, the input, ψ = ∅ − ∅_0_,
- relative frequency of the CAO oscillator w.r.t, the input, Ω = ω −ω_0_, and
- the normalized phase difference between the CAO oscillator and the SRO, $\lambda_{r}=\frac{\emptyset }{\omega^{*}}\;-\;\frac{\emptyset_{r}}{\omega_{r}}$,
  the Equations 9–11 can be simplified to:

(12a) $$\begin{array}{l} \dot{r}\;=\;\left(\mu -\beta_{1}r^{2}\right)r\;+\;A_{r}{r_{r}}^{\frac{\omega^{*}}{\omega_{r}}}\cos\left(\theta_{r}\;-\;\lambda_{r}\omega^{*}\right)\;+\;\varepsilon I_{0}\cos\psi \end{array}$$

(12b) $$\begin{array}{l} \dot{\psi }\;=\;\Omega \;+\;A_{r}\frac{{r_{r}}^{\frac{\omega^{*}}{\omega_{r}}}}{r}\sin\left(\theta_{r}\;-\;\lambda_{r}\omega^{*}\right)\;-\;\frac{\varepsilon I_{0}}{r}\sin\psi \end{array}$$

(13a) $$\begin{array}{l} \dot{r_{r}}\;=\;\left(\mu_{r}\;-\;\beta_{1r}{r_{r}}^{2}\right)r_{r} \end{array}$$

(13b) $$\begin{array}{l} \dot{\emptyset_{r}}=\omega_{r} \end{array}$$

(14) $$\begin{array}{l} \tau_{\omega }\dot{\omega^{*}}\;=\;-\;\omega^{*}\;+\;\omega \;+\;A_{r}\frac{{r_{r}}^{\frac{\omega^{*}}{\omega_{r}}}}{r}\sin\left(\theta_{r}\;-\;\lambda_{r}\omega^{*}\right)\;-\;\frac{\varepsilon I_{0}}{r}\sin\psi \end{array}$$

Under the special condition, 0 < ε ≪ 1, the single unit is equivalent to a pair of unidirectionally coupled oscillators through modified power coupling. There are two differences between the modified power coupling used under the unilateral coupling scenario in this study and the conventional power coupling proposed in (Biswas et al., 2021). In the conventional power coupling scheme of (Biswas et al., 2021), the complex state of the oscillator is raised to the ratio of the *intrinsic* frequencies of the presynaptic and postsynaptic oscillators. In the modified power coupling proposed now, the exponent is the ratio between the *actual* frequency of oscillation of the post-synaptic oscillator and the *actual* frequency of oscillation of the presynaptic oscillator (denoted by the dynamical variable ω^*^ as defined by Equations 11 and 14). The second difference is that, in modified power coupling, the angle of the complex power coupling coefficient does not incorporate the natural frequency of the presynaptic oscillator in the denominator (conventional and modified power coupling coefficients are: $W_{ij}=A_{ij}e^{i\frac{\theta_{ij}}{\omega_{j}}}$ and $W_{ij}=A_{ij}e^{i\theta_{ij}},$ respectively, where *i* and *j* are the indices of the presynaptic and the postsynaptic oscillator, respectively). At a steady state, the normalized phase difference between the two oscillators $\left(\frac{\emptyset }{\omega^{*}}-\frac{\emptyset_{r}}{\omega_{r}}\right)$ will be $\frac{1}{\omega }$ times the angle of the complex coupling coefficient (θ_*r*_) (see Appendix 1 for proof).

**Figure 3:**
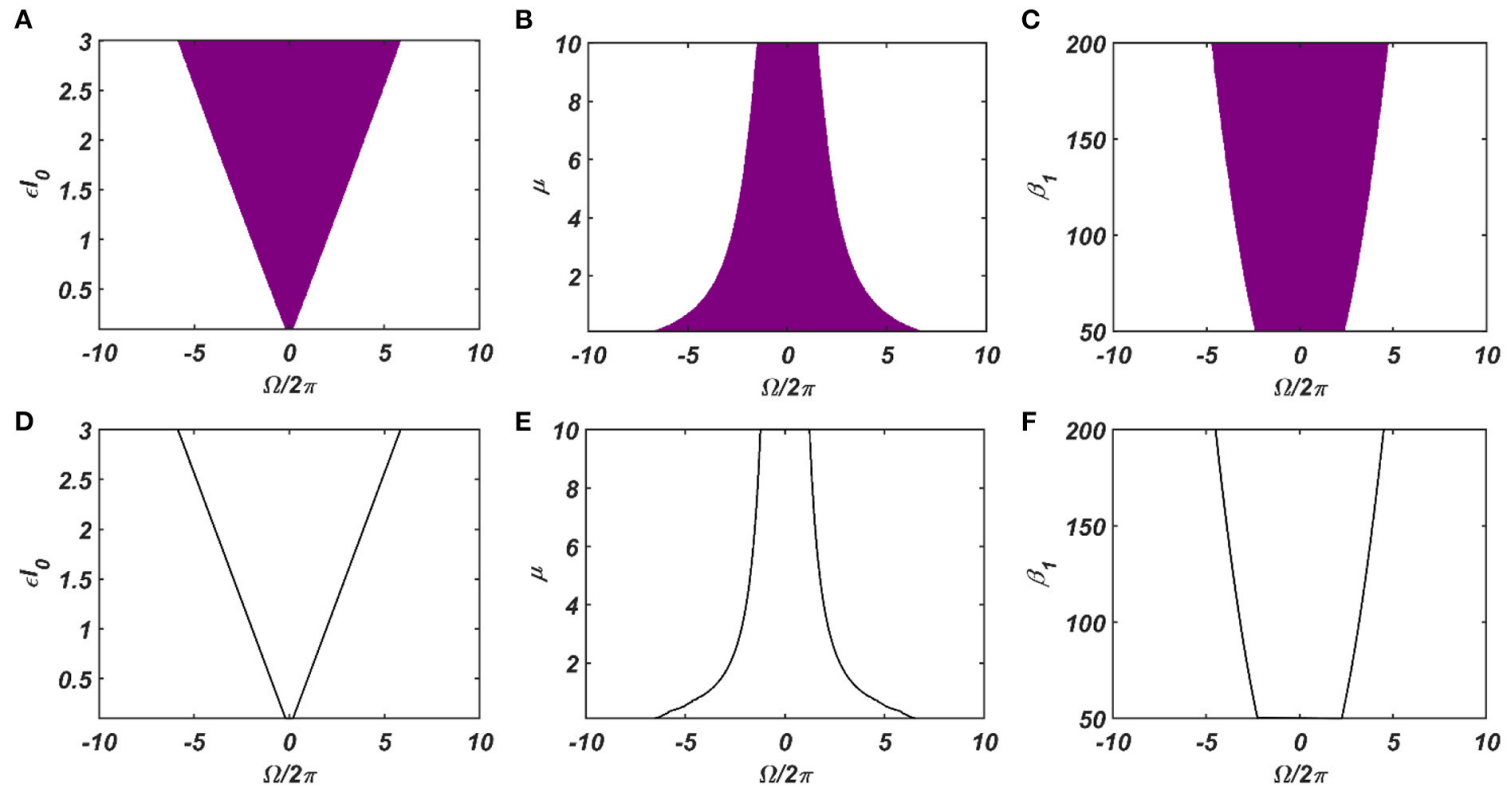
The purple region on the **(A–C)** plots shows the entrainment regime of an individual Hopf oscillator and how it is dependent on its intrinsic dynamical parameters (μ and β_1_) and the intensity of the driving input (ε*I*_0_). Whereas the boundaries of the entrainment regime on this parameter space plotted in **(D–F)**, represented by the function $\frac{\Omega r_{ss}}{\varepsilon I_{0}}=1$. In each of these plots, one of these parameters is varied while keeping others fixed at: μ = 1, β_1_ = 150, ε*I*_0_ = 2.

Under normal condition (ε ≠ 0), we are going to consider the special scenario where ω_0_ falls under the entrainment regime of the CAO oscillator. The entrainment regime of an individual oscillator, receiving only ε*I*(*t*) as input, denotes the range of values of Ω for which the Hopf oscillator exhibits either stable a fixed point or stable spiral behavior in (*r*, ψ) space under the influence of *I*(*t*). Inside entrainment regime, the actual frequency of oscillation (ω^*^) of the oscillator is entrained to the frequency of the input signal (ω_0_) if the natural frequency of the Hopf oscillator (ω) is sufficiently close to ω_0_. (i.e., |Ω| is sufficiently small). In Figure 3, the entrainment regime can be identified as the purple region as a function of μ, β_1_, and the intensity of the driving input (ε*I*_0_). The boundary of the entrainment regime on this parameter space can also be identified as an analytical expression. From Appendix 2, we found out that the steady-state phase offset of the oscillator inside the entrainment regime is: $\xi_{0}+\frac{\Omega r_{ss}}{\varepsilon I_{0}}$. At the boundary of the entrainment regime, the argument of the arcsin operator is 1. i.e., $\frac{\Omega r_{ss}}{\varepsilon I_{0}}=1$, which is presented in Figures 3D–F as a function of one of these three parameters keeping other two fixed.

**Figure 4:**
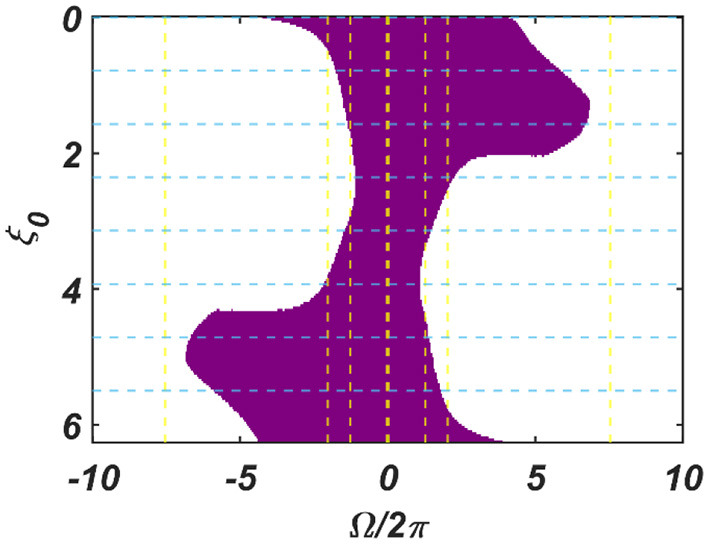
The figure principally depicts the entrainment regime (the purple region) of the CAO oscillator on the Ω vs θ_*r*_ − ξ_0_ plane, keeping the other factors, such as ε*I*_0_, θ_*r*_, *A*_*r*_ and the intrinsic parameters of the CAO oscillator and the SRO, fixed.

The dependency of the width of the entrainment regime, ω on μ, β_1_, and ε*I*_0_, is further illustrated in Figure A2.2 in the Appendix. Although, at the beginning, the CAO oscillator is perturbed by two complex sinusoidal input signals, one with frequency ω (from the SRO, but after the modified power-coupling step) and the other with frequency ω_0_ (external input *I*(*t*)), it is entrained to the external input signal because of the dominance of the perturbation by *I*(*t*) over the perturbation caused by the input from SRO since we assume that *A*_*r*_ < ε*I*_0_. The entrainment width of the CAO oscillator will also depend on θ_*r*_ − ξ_0_ (Figure 4). Since the SRO does not receive any inputs, it always oscillates at its natural frequency of oscillation (ω_*r*_).

**Figure 5:**
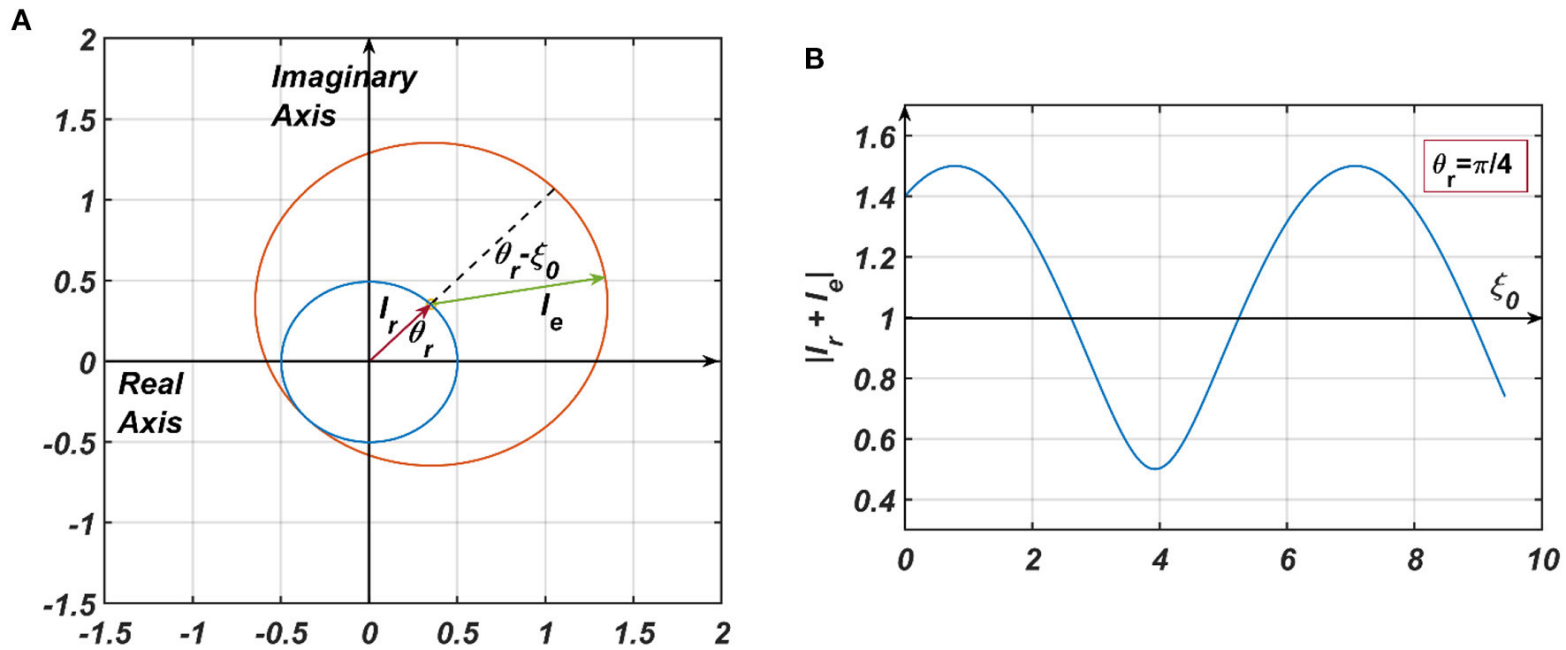
**(A)** The pictorial representation of the constructive and destructive interference between the two inputs to a CAO oscillator. $I_{r}=.5e^{i\theta_{r}}$, $I_{e}=e^{i\xi_{0}}$ are the phasors of the input from the reference oscillator and the external input, respectively. *I*_*t*_ = *I*_*e*_ + *I*_*r*_. In **(B)** θ_*r*_ is kept fixed at $\frac{\pi }{4}$, and ξ_0_ is varied to plot |*I*_*t*_| from which it can be observed that |*I*_*t*_| is maximum when ξ_0_ = 2*nπ* + θ_*r*_ and minimum when ξ_0_ = (2*n* + 1)π + θ_*r*_.

Thus, under the conditions of entrainment, a given CAO oscillator gets two complex sinusoidal signals as inputs with the same frequency as *I*(*t*), with different magnitude and phase offsets, given as follows:

1. the 3^rd^ term in the RHS of Equation 9 evolves to${\;W}_{ij}^{r}{z_{r}}^{\frac{\omega_{ij}^{*}}{\omega_{r}}}=A_{ij}^{r}{r_{r}}^{\frac{\omega_{ij}^{*}}{\omega_{r}}}e^{i\left(\theta_{ij}^{r}+\emptyset_{r}\frac{\omega_{ij}^{*}}{\omega_{r}}\right)}=A_{ij}^{r}{r_{r}}^{\frac{\omega_{0}}{\omega_{r}}}e^{i\left(\theta_{ij}^{r}+\emptyset_{r}\frac{\omega_{0}}{\omega_{r}}\right)}=A_{ij}^{r}{r_{r}}^{\frac{\omega_{0}}{\omega_{r}}}e^{i\left(\omega_{0}t+\theta_{ij}^{r}\right)}$ at a steady state (refer to Appendix 4), denoted by $I_{r}\left(t\right)=a_{r}e^{i\left(\omega_{0}t+\xi_{r}\right)}$ and
2. the 4^th^ term in RHS of Equation 9, $\varepsilon I_{0}e^{i\left(\omega_{0}t+\xi_{0}\right)},$ denoted by $I_{e}\left(t\right)=a_{0}e^{i\left(\omega_{0}t+\xi_{0}\right)}$ (Appendix 4), where *a*_*r*_ is dependent on the steady state magnitude of oscillation of the reference oscillator (*r*_*rss*_), which, in turn, depends on μ_*r*_, β_1*r*_ and *A*_*r*_. ξ_*r*_ being θ_*r*_ justifies why the actual frequency of the presynaptic oscillator is omitted from the denominator of the angle of the modified power coupling coefficient. Both of these inputs either constructively or destructively interfere with each other, depending on their relative phase offsets (ξ_*r*_ − ξ_0_ = θ_*r*_ − ξ_0_). Since the magnitude of the response of the CAO oscillator is either diminished or increased, depending on the relative values of θ_*r*_ and ξ_0_, with this arrangement, the CAO oscillator will be capable of encoding the phase offset of the complex sinusoidal input signal (qualitatively portrayed in Figure 5, later elaborated in the *Second stage of training: Training phase offset Subsection of the Results* section).

### Modified Hebbian learning

A modified Hebbian learning rule is proposed for training the modified power coupling described in the previous section. The complex variable and the polar coordinate version of the modified Hebbian learning rule are described as follows:


(15) $$\begin{array}{l} \tau_{W}\dot{W_{r}}\;=\;-\;W_{r}\;+\;z{\left(\bar{z_{r}}\right)}^{\frac{\omega^{*}}{\omega_{r}}} \end{array}$$

(15a) $$\begin{array}{l} \tau_{W}\dot{A_{r}}\;=\;-\;A_{r}\;+\;r{r_{r}}^{\frac{\omega^{*}}{\omega_{r}}}\cos\omega^{*}\left(\frac{\emptyset }{\omega^{*}}\;-\;\frac{\theta_{r}}{\omega^{*}}\;-\;\frac{\emptyset_{r}}{\omega_{r}}\right) \end{array}$$

(15b) $$\begin{array}{l} \tau_{W}\dot{\theta_{r}}\;=\;\frac{r{r_{r}}^{\frac{\omega^{*}}{\omega_{r}}}}{A_{r}}\sin\omega^{*}\left(\frac{\emptyset }{\omega^{*}}\;-\;\frac{\theta_{r}}{\omega^{*}}\;-\;\frac{\emptyset_{r}}{\omega_{r}}\right) \end{array}$$

The modified Hebbian learning rule as prescribed in Equations 15a,b can be compared to the original Hebbian learning for the power coupling coefficient proposed earlier (Equations 15 in Biswas et al., 2021). Here, $\bar{z_{r}}$ is the complex conjugate of the complex activation of the SRO. It can be observed that the modified Hebbian learning rule for the modified power coupling coefficient has a similar effect as the original Hebbian rule had in case of the previously proposed power coupling strategy.

Effectively, the modified Hebbian learning rule is the same as the previous one when there is no entrainment. Without entrainment when the phase offset of the post-synaptic oscillator is driven to the phase offset of the complex sinusoidal input signal with identical frequency as the natural frequency of the CAO oscillator, θ_*r*_ learns ξ_0_, where ξ_0_ is the phase offset of the input driving the post-synaptic oscillator (refer to Appendix 5). Similarly, it can be shown that *A*_*r*_ learns $r_{ss}{r_{rss}}^{\frac{\omega }{\omega_{r}}}$ (refer to Appendix 6), where *r*_*ss*_ and *r*_*rss*_ are the steady state values of amplitude of oscillation of the post-synaptic and presynaptic oscillators, respectively. With entrainment of the main oscillator, θ_*r*_ learns $\xi_{0}+\frac{\Omega r_{ss}}{\varepsilon I_{0}}$, which is the phase offset of the main oscillator at a steady state after entrainment, while the Hebbian dynamics is enabled.

### Training the OTSOM

In the previous section, we discussed the response properties of a single CAO oscillator to its two inputs. We now discuss the training methodology of OTSOM. The OTSOM model is trained on complex sinusoidal signals, each defined by a characteristic frequency (ω_0_) and phase (ξ_0_) (defined w.r.t. SRO). Thus, for a given sinusoidal input, we expect the OTSOM to produce a single winner, such that the row number, *i*, of the winner represents the input phase, while the column number, *j*, represents the input frequency. Therefore, the CAO oscillator at (*i, j*) gives maximum resonating response when ω_0_ = ω_*ij*_ and $\xi_{0}=\theta_{ij}^{r},$ which is why we have chosen to train the ω_*ij*_ and $\theta_{ij}^{r}$ parameters of the OTSOM network. These parameters are trained in two consecutive training stages. In the 1st stage, ω_*ij*_s are trained keeping $\theta_{ij}^{r}$'s fixed, and, in the 2^nd^ stage, $\theta_{ij}^{r}$s are trained by keeping the already trained ω_*ij*_s fixed.

### First stage of training: Training frequency

In this stage, the natural frequencies, ω_*ij*_, of the CAO oscillators are trained according to a learning rule analogous to the self-organizing map learning rule (Kohonen, 1990). Specifically, to train the frequencies of the individual oscillators, we used the adaptive frequency Hopf oscillator model proposed earlier (Righetti et al., 2005; Biswas et al., 2021). The training takes place over multiple epochs (*N*_*epoch*, ω_). In every epoch, *N* input signals are randomly chosen from the input set *Y*. The input set contains complex sinusoidal signals with frequencies and phase offsets sampled from a uniform probability distribution. Once an input signal with certain frequency and phase offsets ($I_{0}e^{i\left(\omega_{0p}t+\xi_{0p}\right)}$) is selected, it is presented as the external input signal (*I*_*p*_(*t*)) to the oscillators in the CAO. After an initial transient phase, which lasts for *T*_*sω*_ seconds, the CAO oscillator response reaches a steady state. The oscillator with the largest amplitude at the end of the transient phase is denoted the “winner CAO oscillator”.

The dynamics of Equations (9–11) is simulated with the special condition $A_{ij}^{r}≅0$ during the transient phase. Under this condition, the steady state solution of CAO oscillator ($r_{ijss}^{*}$, $\psi_{ijss}^{*}$) can either be a stable node, stable spiral or unstable spiral (Kim and Large, 2015). Typically, inside the entrainment regime, we get a stable node or stable spiral as the solution. The parameters μ, β_1_, and ε play a crucial role during this phase as they determine not only the entrainment width of the Hopf oscillator (ω) but also the duration of the transient period (*T*_*sω*_). As the oscillator with the highest steady state amplitude of oscillation (max(*r*_*ijss*_)) is chosen to be the winner, it is evident that the winner oscillator should satisfy the condition |ω_*ij*_ − ω_0*p*_|. After the transient period, the frequencies ω_*ij*_s of all the CAO oscillators within the neighborhood window, along with the winner oscillator, are trained for *T*_*tω*_ secs according to the following dynamics (Biswas et al., 2021):


(16) $$\begin{array}{l} \dot{\omega_{ij}}\;=\;-\;{\eta_{\omega }}_{ij}^{mn}\left(t\right)\varepsilon I_{0}\sin\psi_{ij} \end{array}$$

where ${\eta_{\omega }}_{ij}^{mn}$ is the neighborhood function, centered on the winner oscillator located on the *m*^*th*^ row and the *n*^*th*^ column, defined as the following:


$$\begin{array}{l} \eta_{\omega }{\;}_{ij}^{mn}(t)=W_{ij}^{mn}\eta_{\omega }{\;}_{0}e^{\;-\;\frac{{\left(i-m\right)}^{2}}{\sigma_{y\omega }\left(t\right)}\;-\;\frac{{\left(j-n\right)}^{2}}{\sigma_{x\omega }\left(t\right)}}\\ \sigma_{y\omega }\left(t\right)=\sigma_{y\omega m}e^{-\;\frac{i_{epoch}^{2}}{\sigma_{\sigma y\omega }}}\\ \sigma_{x\omega }\left(t\right)=\sigma_{x\omega m}e^{-\;\frac{i_{epoch}^{2}}{\sigma_{\sigma x\omega }}}\\ W_{ij}^{mn}=1\;\in \left|i-m\right|\leq d_{r\omega }\;and\;\left|j-n\right|\leq d_{c\omega }\\ \;=0\;otherwise \end{array}$$

$W_{ij}^{mn}$ is termed as the neighborhood window. Here, *d*_*rω*_ and *d*_*cω*_ are the half-lengths of the neighborhood window along rows and columns, respectively. Effectively, the purpose of the neighborhood window is to constrain the learning confined to the neighborhood of the winner oscillator. Additionally, it can be observed that the neighborhood function, ${\eta_{\omega }}_{ij}^{mn}$, is a Gaussian centered on the winner oscillator. The standard deviations along the rows and columns (σ_*yω*_ and σ_*xω*_) of ${\eta_{\omega }}_{ij}^{mn}$ decrease with time to produce annealing effect. Thus, the network dynamics is simulated for (*T*_*sω*_ + *T*_*tω*_) seconds for each presentation of a training signal, for *N*(*T*_*sω*_ + *T*_*tω*_) during each training epoch, where N is the number of training signals. Therefore, the time required for the entire first phase of training is *N*_*epoch*, ω_*N*(*T*_*sω*_ + *T*_*tω*_).

### Second stage of training: Training phase off-set

While the objective of the first stage training is to train the frequencies, ω_*ij*_s, of the CAO oscillators, the objective of the second stage training is to train the phases of the same oscillators which are determined by the angles, $\theta_{ij}^{r}$, of feedforward connections from the SRO to the CAO oscillators. After the first-stage training, ω_*ij*_s self-organize in an increasing order along the rows, a pattern confirmed by the simulations shown in the results section. This occurs because the CAO is a rectangular array with the number of columns much larger than the number of rows. The 2nd stage of training commences with randomly initialized $\theta_{ij}^{r}$ parameters and follows the algorithmic course as given in Figure 5B.

In the second-stage training, during the transient period, the magnitudes of $A_{ij}^{r}$ parameters are increased to the same order of magnitude as ε. The training takes place through multiple epochs (*N*_*epoch*, θ_) in which, as in the previous stage, each training pattern is a complex sinusoidal signal with specific ω_0*p*_ and ξ_0*p*_, sampled from the input set *Y*. As in the previous training stage, the network dynamics is first allowed to reach the steady state before $\theta_{ij}^{r}$s are adapted. The transient phase dynamics, given by Equations 9–11, is simulated for *T*_*sθ*_ secs. At the end of the transient period, the winner oscillator is identified by its steady state amplitude of oscillation (r_*ijss*_). It is expected that the natural frequency (ω_*mn*_) and the angle of the power coupling coefficient ($\theta_{mn}^{r}$) of the winner oscillator should be closest to ω_0*p*_ and ξ_0*p*_, respectively. During the subsequent *T*_*hθ*_ period, $\theta_{ij}^{r}$ parameters of the unidirectional power coupling coefficients are trained according to the learning rule given in eqn-17. As eqn-17 is derived from the modified Hebbian learning rule as proposed in eqn-15b, this phase is termed as the Hebbian learning phase.


(17) $$\begin{array}{l} \dot{\theta_{ij}^{r}}\;=\;{\eta_{\theta }}_{ij}^{mn}\left(t\right)\frac{r_{ij}{r_{r}}^{\frac{\omega_{ij}^{*}}{\omega_{r}}}}{A_{ij}^{r}}\sin\omega_{ij}^{*}\left(\frac{\emptyset_{ij}}{\omega_{ij}^{*}}\;-\;\frac{\theta_{ij}^{r}}{\omega_{ij}^{*}}\;-\;\frac{\emptyset_{r}}{\omega_{r}}\right) \end{array}$$

$$\begin{array}{l} {\eta_{\theta }}_{ij}^{mn}\left(t\right)\;=\;W_{ij}^{mn}\left(\left({\eta_{\theta }}_{\max}\;-\;{\eta_{\theta }}_{\min}\right)e^{\;-\;\frac{{\left(i-m\right)}^{2}}{\sigma_{y\theta }\left(t\right)}\;-\;\frac{{\left(j-n\right)}^{2}}{\sigma_{x\theta }\left(t\right)}}\;+\;{\eta_{\theta }}_{\min}\right)\\ \sigma_{y\theta }\left(t\right)\;=\;\sigma_{y\theta m}e^{\;-\;\frac{i_{epoch}^{2}}{\sigma_{\sigma y\theta }}}\\ \sigma_{x\theta }\left(t\right)\;=\;\sigma_{x\theta m}e^{\;-\;\frac{i_{epoch}^{2}}{\sigma_{\sigma x\theta }}} \end{array}$$

$$\begin{array}{l} W_{ij}^{mn}\;=\;1\;\in |i-m|\leq d_{r\theta }\;and\;|j-n|\leq d_{c\theta }\\ =\;0\;otherwise \end{array}$$

Here, *m* and *n* denote the index of the winner oscillator. The Gaussian shaped neighborhood function, ${\eta_{W}}_{ij}^{mn}$, is confined between η_*Wmax*_ and η_*Wmin*_. The learning neighborhood window $W_{ij}^{mn}$ is defined w.r.t, the winner oscillator similar to the first stage of training except that *d*_*rθ*_ and *d*_*cθ*_ are the half-lengths of the neighborhood window. In the Hebbian learning phase, $A_{ij}^{r}$ parameters are not trained ($\dot{A_{ij}^{r}}=0$); we have kept $A_{ij}^{r}$ values close to zero or $A_{ij}^{r}\ll \varepsilon I_{0}$. In which case, $\theta_{ij}^{r}$ learns $\xi_{0p}+\frac{\Omega_{ij}^{p}r_{ij,ss}}{\varepsilon I_{0}}$ at a steady state when the CAO oscillator receives the input $I_{0}e^{i\left(\omega_{0p}t+\xi_{0p}\right)}$ (refer to Appendix 5) (where $\Omega_{ij}^{p}=\omega_{ij}-\omega_{0p}$). It has also been shown that, even if $A_{ij}^{r}$ is not negligible (i.e., $\dot{A_{ij}^{r}}=0,\;A_{ij}^{r}\neq 0$) $\theta_{ij}^{r}$ learns $\xi_{0p}+\frac{\Omega_{ij}^{p}r_{ij,ss}}{\varepsilon I_{0}}$ at the steady state (refer to Appendix 6). This happens because the phase offset of the CAO oscillator (δ_*ij*_) attains the value $\xi_{0p}+\frac{\Omega_{ij}^{p}r_{ij,ss}}{\varepsilon I_{0}}$ at the steady state inside the entrainment regime. Each training epoch in the 2^nd^ stage training takes *N*(*T*_*sθ*_ + *T*_*hθ*_) seconds, and the time required for the entire 2^nd^ stage of training is *N*_*epoch*, θ_*N*(*T*_*sθ*_ + *T*_*hθ*_).

## Results

### Single oscillator results

We now numerically analyze the response of a single CAO oscillator to the simultaneously received inputs from SRO [(*t*))] and the external input [(*t*))] for the following conditions:

(*a*)*A*_*r*_ being sufficiently smaller but not negligible w.r.t ε*I*_0_ [i.e., the strength of the *I*_*e*_(*t*))], which enables the CAO oscillator to get entrained to *I*(*t*), and ensures significant interference between the two inputs. The condition can be mathematically rephrased as:


(18a) $$\begin{array}{l} \epsilon_{\max}\;>\;\varepsilon I_{0}\;-\;A_{r}\;>\;\epsilon_{\min} \end{array}$$

where ϵ_min_ and ϵ_max_ are positive numbers.

(*b*)*A*_*r*_ is negligible w.r.t ε*I*_0_, which is similar to the scenario where CAO oscillator is only perturbed by *I*(*t*).


(19a) $$\begin{array}{l} A_{r}\;\ll \varepsilon \;I_{0} \end{array}$$

**Figure 6:**
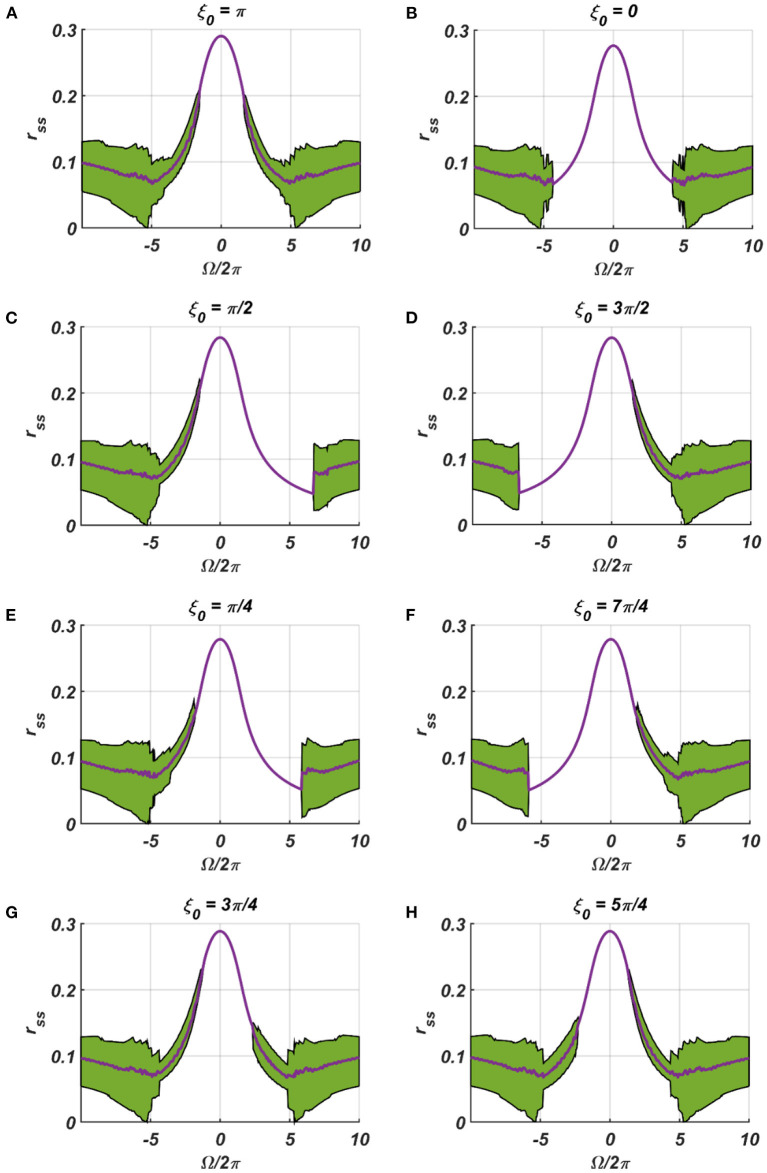
**(A–H)** denotes the steady-state magnitude of oscillation of the CAO oscillator with the identical pair of coupled CAO oscillator and SRO as provided in the Figure 4 but for a symmetrical distinct value of the phase offset of the external input signal w.r.t the angle of the power coupling connection θ_*r*_ = π, cross section of which is drawn in blue lines in Figure 4. The green region depicts the variance of steady state magnitude of oscillation w.r.t the mean value, plotted in purple.

**Figure 7:**
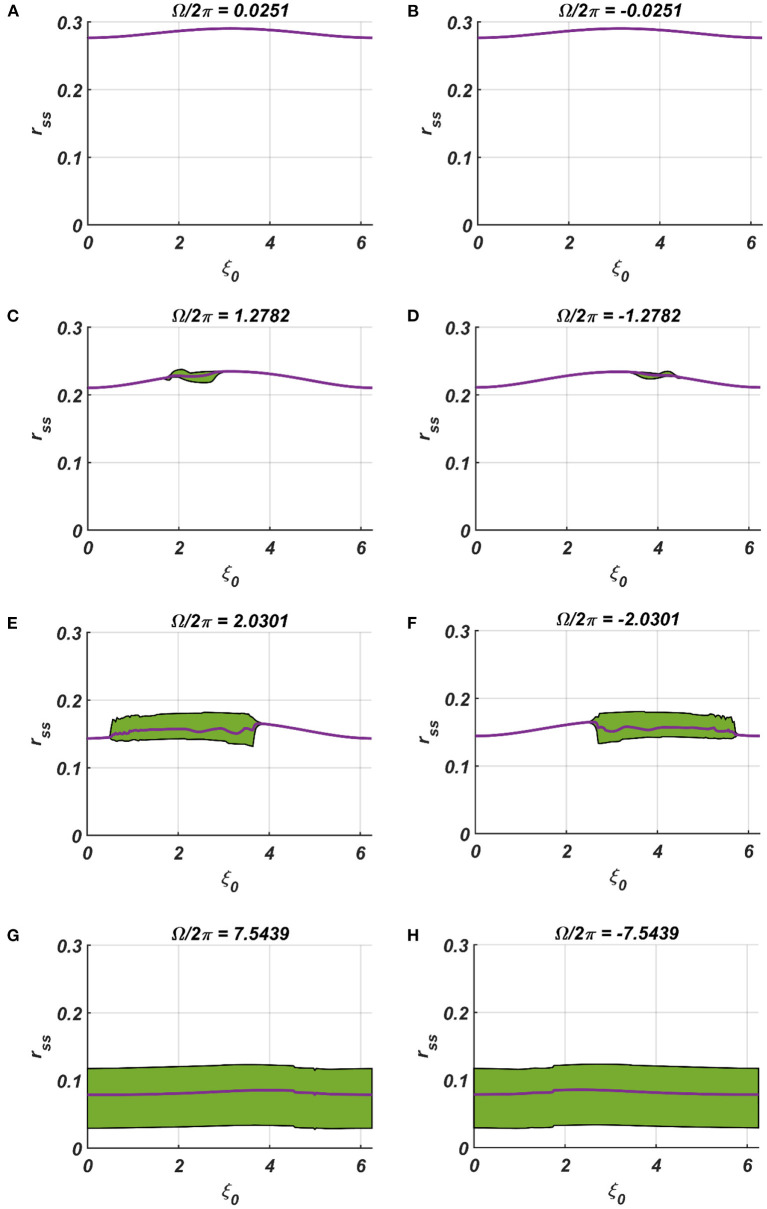
**(A–H)** Denotes the steady-state magnitude of oscillation of the CAO oscillator with the identical pair of coupled CAO oscillator and SRO as provided in the Figure 4 but for a symmetrical distinct value of the relative frequencies of the external input signal w.r.t the natural frequency of the CAO oscillator ω = 2π × 60, cross section of which is drawn in yellow lines in Figure 4. The green region depicts the variance of steady state magnitude of oscillation w.r.t the mean value, plotted in purple.

**Figure 8:**
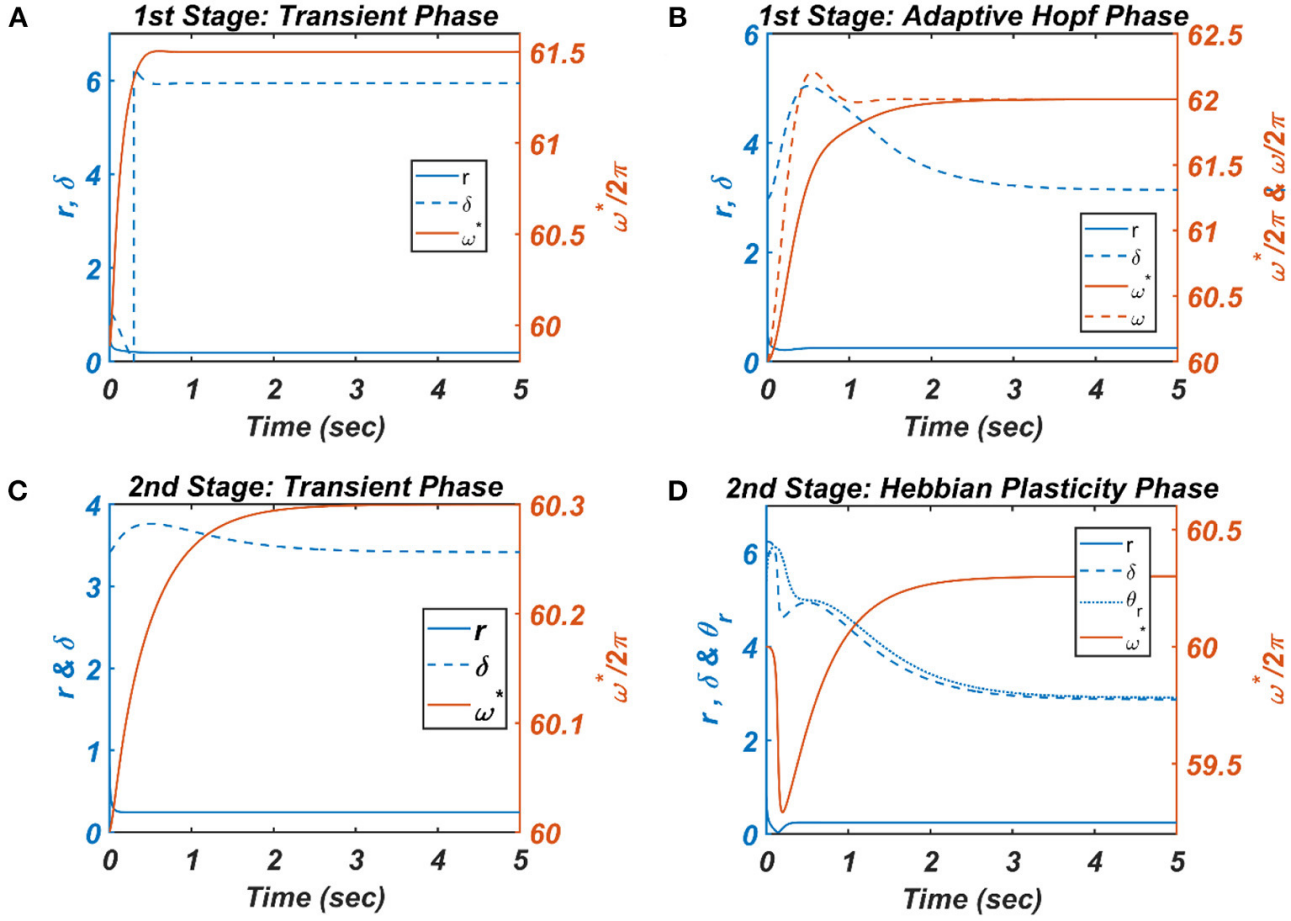
The figure depicts the response of the CAO oscillator in terms of its magnitude of oscillation (*r*), phase offset (δ), entrainment (ω^*^) during all the four phases of the 1^st^ and 2^nd^ stages of the training along with the parameters to be trained (ω and θ_*r*_) and the other fixed parameters μ = 1, β_1_ = 150, ε = 2, *I*_0_ = 1, μ_*r*_ = 1, β_1*r*_ = 10, ω_*r*_ = 2π × 60.5. **(A)** During the transient phase of the 1^st^ stage of the training, the single unit is simulated under the entrainment regime of the CAO oscillator with the additional parameters: ω_0_ = 2π × 61.5,$\xi_{0}=\frac{\pi }{4}$,θ_*r*_ = π, $\tau_{\omega }=8\times 10^{-4}$, $A_{r}=10^{-5}$. It can be verified that at steady-state *r* and δ attain the solution provided by Equation A1.8 and the value $\xi_{0}+\frac{\Omega r_{ss}}{\varepsilon I_{0}}$, respectively. **(B)** For the adaptive Hopf phase of the 1^st^ stage of the training, the single unit is simulated with the additional parameters: ω(0) = 2π × 60, ω_0_ = 2π × 62,ξ_0_ = π,θ_*r*_ = π, τ_ω_ = 0.5, $A_{r}=10^{-5}$, η_ω_ = 50. It can be verified that, at a steady state, δ attains ξ_0_ and ω learns ω_*o*_. **(C)** The single unit is simulated with the following set of additional parameters during the transient phase of the 2^nd^ stage of the training: ω_0_ = 2π × 60.3,ξ_0_ = 3.6773, τ_ω_ = 0.5, *A*_*r*_ = 0.1. The steady-state values of *r* depend on $|{\bar{F}}_{net}|,$ whereas the steady-state value of δ is the same as $\arg\left({\bar{F}}_{net}\right)$. **(D)** For the Hebbian plasticity phase of the 2^nd^ stage of the training, the single unit is simulated with the additional parameters: ω_0_ = 2π × 60.3,ξ_0_ = π, τ_ω_ = 0.5, $A_{r}=10^{-5}$, $\eta_{\theta }=10^{-5}$. It can be verified that θ_*r*_ learns $\xi_{0}+(\frac{\Omega r_{ss}}{\varepsilon I_{0}}),$ which is the same as the steady-state value of δ.

We have numerically analyzed the response of the CAO oscillator under the 1^st^ condition (Equation 18a) as described by the Equations 9–11. The CAO oscillator exhibits stable entrainment, depending on both the relative frequency (Ω) and the relative phase (θ_*r*_ − ξ_0_) w.r.t, the external input signal. When it shows stable entrainment, the magnitude of oscillation reaches a fixed value at a steady state. There is a distinguishable boundary on the Ω vs θ_*r*_ − ξ_0_ plane in which stable entrainment is observed. Outside this boundary, the CAO either exhibits intermittent entrainment or does not get entrained at all. In the following figure (Figure 4), the region in the Ω vs θ_*r*_ − ξ_0_ plane where stable entrainment is observed is portrayed as the purple region. It can be observed that there is a symmetry in the purple region w.r.t the Ω = 0 and θ_*r*_ − ξ_0_ = 0 axis (θ_*r*_ = π). A pair of CAO oscillator and an SRO is simulated until the steady state is achieved to find out the mean, maximum, and the minimum values of the steady state magnitude of oscillation of the CAO oscillator. The following parameters are used: μ = 1, β_1_ = −100, ω = 2π60, ω_0_ = 2π50 *to* 2π70, ξ_0_ = 0 *to* 2π, ε = 2, *F* = 1, μ_*r*_ = 1, β_1*r*_ = −10, ω_*r*_ = 2π × 60, τ_ω_ = 0.1, *A*_*r*_ = 0.5, θ_*r*_ = π for the simulation results provided in the figures (Figures 6–8). The steady state is attained typically before 3 s. The steady state behavior of the magnitude of oscillation of the CAO oscillator for eight different symmetrical cross sections parallel to Ω = 0 axis and for another eight different symmetrical cross sections parallel to θ_*r*_ − ξ_0_ = 0 axis is plotted in the Figures 6, 7.

In the Figures 7, 8 the *r*_*ss*_ is plotted on a same scale of magnitude so that the resonance exhibited by the CAO oscillator w.r.t, the frequency and the phase offset of the external input, can be compared. It is apparent that ω has a greater effect on the CAO oscillator in terms of its steady-state magnitude of oscillation than ξ_0_. The reason behind introducing the lower bound on ε*I*_0_ − *A*_*r*_ (ϵ_min_) is justified here. In other words, the resonance exhibited by the CAO oscillator w.r.t, the ξ_0_ at a smaller scale, is ensured by the condition ε*I*_0_ − *A*_*r*_ > ϵ_min_. The combined results of Figures 6–8 reveal that the CAO oscillator will oscillate with maximum magnitude at a steady state when |Ω| × |θ_*r*_ − ξ_0_| is minimum.

Considering that the CAO oscillator is operating in the entrainment regime under the 1st condition (Equation 18a) and gets entrained to *I*(*t*), the magnitude and the phase offset of oscillation at a steady state are analytically derived in Appendix 4. As the Hopf oscillator always maintains the same phase offset as the phase offset of the complex sinusoidal input signal, the phase offset of the CAO oscillator becomes the phase offset of the resultant input ($angle\left({\bar{F}}_{net}\right)$), obtained by combining the external input, and the input from the SRO through the complex power coupling connection (elaborated in Appendix 4).


$$\begin{array}{l} {\bar{F}}_{net}\;=\;\varepsilon Fe^{i\xi_{0}}\;+\;A_{r}{\frac{\mu_{r}}{\left|\beta_{1r}\right|}}^{\frac{\omega_{0}}{2\omega_{r}}}e^{i\left(\theta_{r}\;+\;\emptyset_{r}\left(0\right)\frac{\omega_{0}}{\omega_{r}}\right)} \end{array}$$

**Figure 9:**
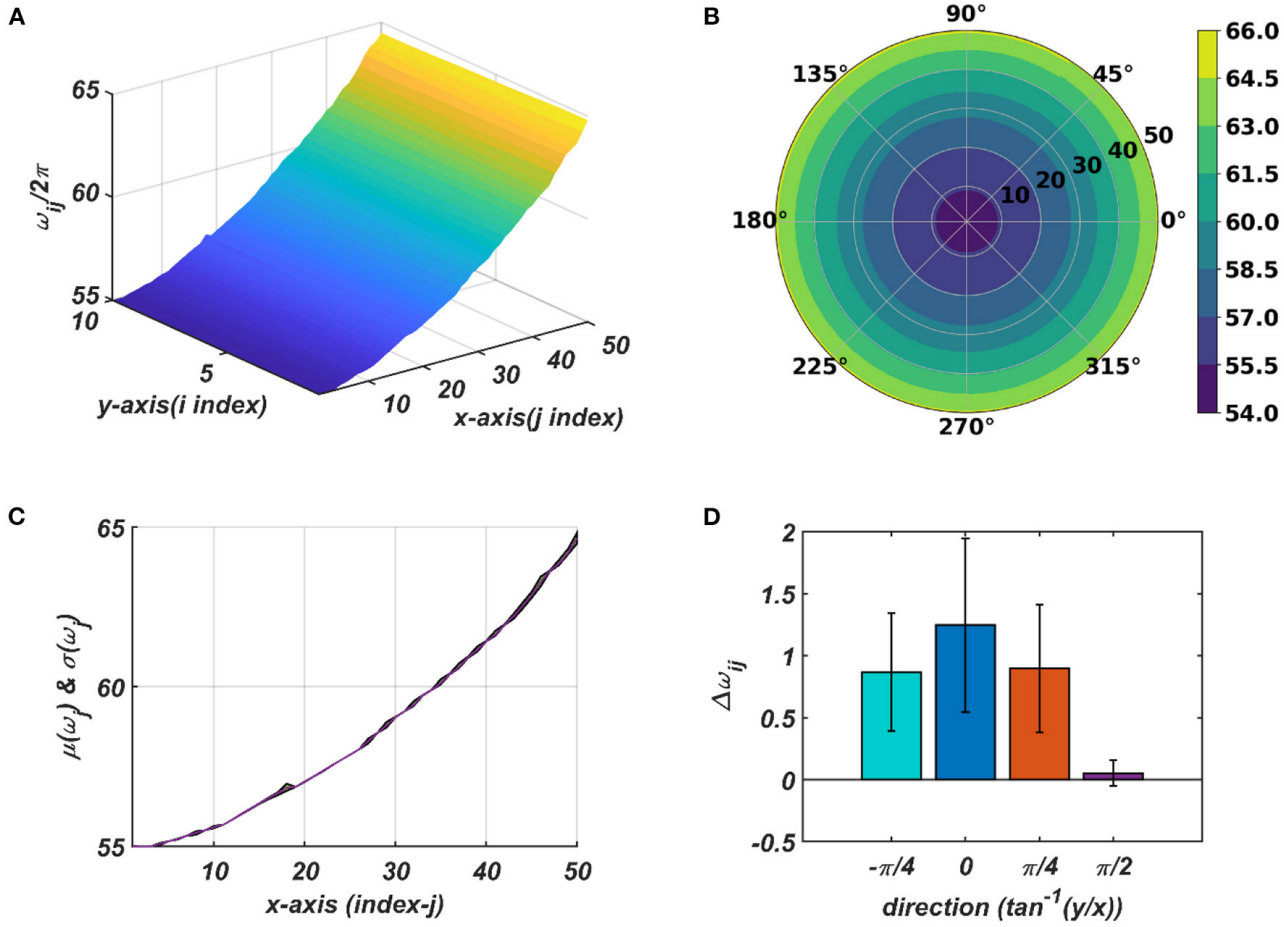
The natural frequency of oscillation, ω_*ij*_, of the oscillators in the CAO after self-organization represented in Cartesian **(A)** and polar **(B)** organization. **(C)** shows mean and the variance of the natural frequencies along x-axis after training. **(D)** provides a quantitative understanding of the slope of self-organized ω_*ij*_s along the x-axis, y-axis, and the axes $\frac{\pi }{4}$ and $-\frac{\pi }{4}$ inclined w.r.t, the x-axis.

The first term on the right-hand side is the phasor of the external input [(*I*(*t*))] through afferent weight, and the second term is the phasor of the input from the SRO through modified power coupling. Note that the magnitude of the resultant input after constructive/destructive interference, $\left|{\bar{F}}_{net}\right|$, determines the magnitude of oscillation of the CAO oscillator at a steady state. Figure 9C numerically verifies that the CAO oscillator attains the analytically derived solutions in Appendix 4.

The effect of μ, β_1_, and ε*I*_0_ on the entrainment width (ω) and the typical transient time (*T*_*t*_) of the CAO oscillator for the 2nd condition (Equation 18b) is further verified numerically. From Figure A2.2 in Appendix 2, it can be verified that μ has to be small for wider entrainment regime, i.e., higher values of μ causes shrinking of the entrainment regime. On the contrary, ε and β_1_ have nearly the same effect on the width of the entrainment regime: entrainment regime broadens as ε and β_1_ values are increased. Since the transient dynamics determines the amount of time the network takes to settle down so that the oscillator with the highest resonant response can be chosen, it is necessary to understand the effect of these parameters on the transient dynamics. It is quite intuitive that μ and β_1_ have a shrinking effect on the transient period as it causes a much steeper basin of attraction around the steady state solution. Figures A2.2D and A2.2F in Appendix show that increasing the magnitude of the scalar afferent weight, ε, shortens the transient period; this is natural because a stronger input pushes the oscillator output to settle down faster. Ideally, the model requires a broad entrainment regime with a short transient period.

### Network level results

#### First stage of training: Training frequency

We may recall from the previous section that the objective of the 1st stage of training is to train the frequencies of the CAO oscillators. To this end, only the external input signals are considered, and the inputs from SRO are ignored (i.e., *A*_*r*_ ≪ ε in Equations 9–11). Initially, the set of the external input signals is constructed by combining input frequency set *Y*_ω_, constructed by sampling *N*_ω_ number of intrinsic frequencies $\left(f_{op}=\frac{\omega_{op}}{2\pi }\right)$ from a uniform distribution over the range of 55 to 65 Hz, and the input phase offset set *Y*_ξ_, constructed by sampling *N*_ξ_ number of phase offset angles (ξ_0*p*_) from a uniform distribution over [0, 2π). Combining these sampled intrinsic frequencies and phase offsets, *N* = *N*_*f*_ × *N*_ξ_ number of complex sinusoidal signal patterns [($I_{p}\left(t\right)=I_{0}e^{i\left(\omega_{0p}t+\xi_{0p}\right)}$)] is generated. After *I*_*p*_(*t*) is chosen randomly from *Y*, the input patterns are presented one at a time to all the oscillators in CAO. Note that, in this case, the winning CAO oscillator depends only on the condition |ω_*ij*_ − ω_0*p*_|, and not on the phase offset, ξ_0*p*_, since *A*_*r*_ ≪ ε*I*_0_.

For each presentation of the input, the winner oscillator and the oscillators in its neighborhood adjust their “preferred frequency” closer to the input frequency, ω_0*p*_, following the adaptive Hopf learning rule of Equation 16. The preferred frequency of the oscillators in the neighborhood of the winner oscillator gets closer to ω_0*p*_ compared to the oscillators near the periphery of the adaptive neighborhood because of the Gaussian neighborhood function, ${\eta_{\omega }}_{ij}^{mn}$. The transient response of the CAO oscillator of a single unit in terms of its magnitude of oscillation (*r*), the phase offset (δ), entrainment (ω^*^) during the transient phase is plotted in Figure 8A, where the analytically derived solutions of *r* and δ (refer to Appendix 2) are verified numerically. Similarly, the dynamic response of the same CAO oscillator in the given single unit in terms of *r*, δ, ω^*^, and ω during the adaptive Hopf phase is presented in Figure 8B, where the analytically derived solutions of *r*, δ, and ω (refer to Appendix 3) are verified numerically.

The natural frequencies of the CAO oscillators are initialized from a uniform random distribution confined to the interval 2π[55, 65]. The natural frequency of the reference oscillator is set to the central frequency (2π60) of the given frequency band so that there is a symmetrical influence by the reference oscillator on CAO oscillators. The size of the CAO (*N*_*r*_, *N*_*c*_) = (10, 50), where *N*_*r*_ is the number of rows and *N*_*c*_ is the number of columns. The adaptive neighborhood is defined by immediate proximity rather than the physical proximity, i.e., a neighborhood size of *d*_*W*_ = 2 means the immediate 2 oscillators on every side of the winner oscillator, including the oscillators situated diagonally.

The tonotopic arrangement emerges at the end of the 1st stage of the training. It can be observed from Figure 9 that ω_*ij*_s organize themselves in an increasing order along *x*-direction or an increasing column index but remain almost invariant along the orthogonal (row) direction. This emergent tonotopic organization is the key feature of a self-organizing map. The parameter values defining the network architecture, the training data set, and the 1st and the 2nd phases of the training are given in Table 1.

**Table 1:** Essential parameters.

**Table 1A**		**Table 1B**		**Table 1C**		**Table 1D**	
**Network and**	**Values**	**Training**	**Values**	**1st stage**	**Values**	**2nd stage**	**Values**
**simulation parameterss**		**set**		**parameters**		**parameters**	
*N*_*x*_	50	*U*_ω_	ω_0*p*_ ~ *U*(55 × 2π, 65 × 2π)	*N*_*epoch*, ω_	500	*N*_*epoch*, ξ_	300
*N*_*y*_	10	*U*_ξ_	ξ_0*p*_ ~ *U*(0, 2π)	*T*_*sω*_	3 s	*T*_*sθ*_	3 s
ε	2	*N*_ω_	60	*T*_*tω*_	4 s	*T*_*hθ*_	4 s
*dt*	0.1 *ms*	*N*_ξ_	10	_η_ω_0_	0.2	_η_θ_max_,_η_θ_min_	2 × 10^−6^, 10^−7^
$A_{ij}^{r}$	10^−5^	*N*= *N*_ω_*N*_ξ_	600	σ_*yωm*_	100	σ_*yθm*_	2
ω_*r*_	60 × 2π			σ_*xωm*_	4	σ_*xθm*_	2
μ	1			σ_σ*xω*_	500 × *n*_*eph*, ω_	σ_σ*xθ*_	∞
μ_*r*_	1			σ_σ*yω*_	500 × *n*_*eph*, ω_	σ_σ*yθ*_	∞
β_1_	150			*d*_*rω*_	3	*d*_*rθ*_	3
β_1*r*_	10			*d*_*cω*_	3	*d*_*cθ*_	3
				$A_{ij}^{r}$	10^−5^	$A_{ij}^{r}$	0.1 in transient phase and 10^−5^ in Hebbian plasticity phase

(A) The table summarizes the list of parameters defining the network architecture, the intrinsic dynamical parameters, and simulation parameters. (B) The details of the training data set are given. The parameters for the 1st and the 2nd stages of the training are listed in (C,D), respectively.

#### Second stage of training: Training phase offset

**Figure 10:**
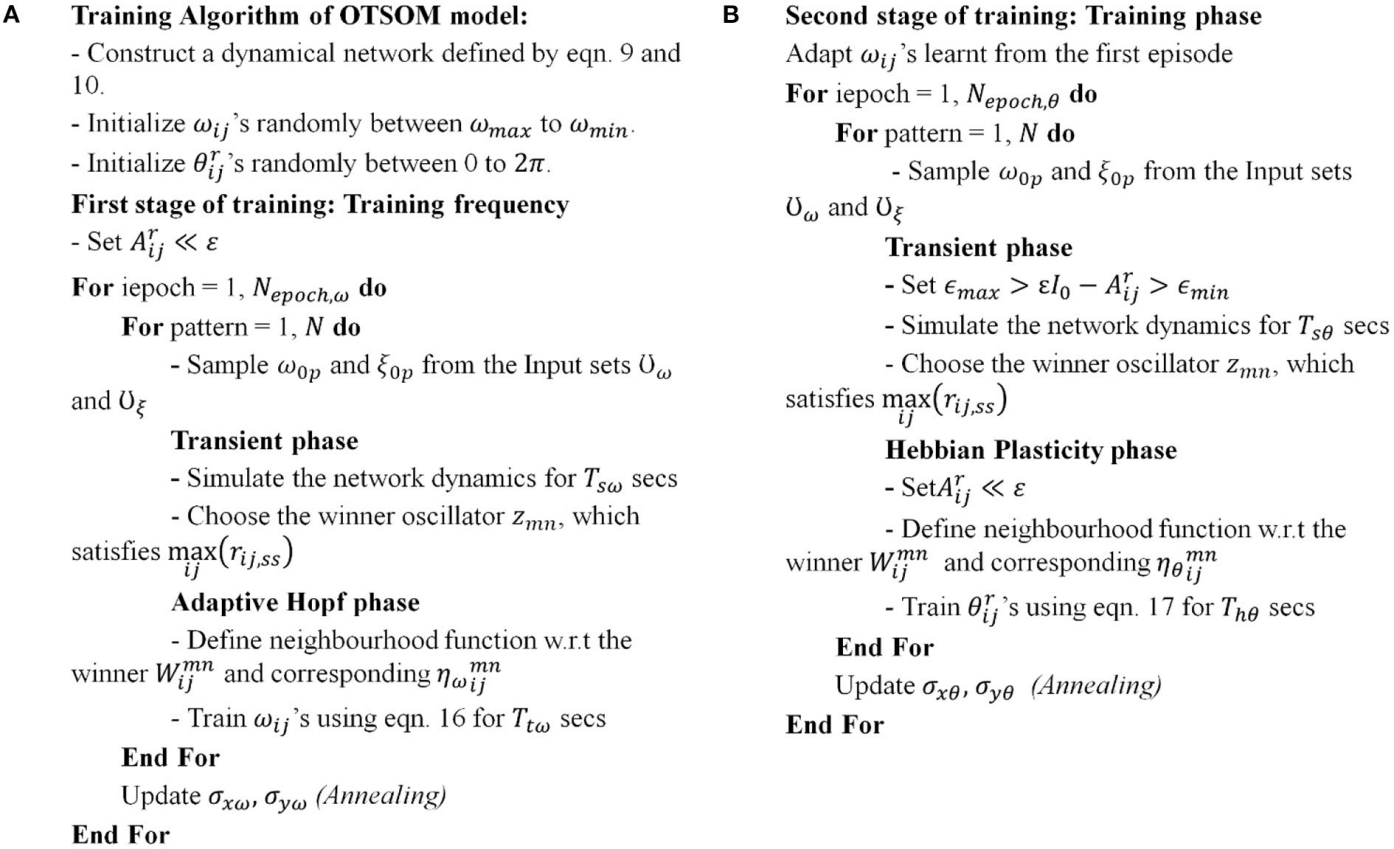
Algorithms for the first **(A)** and the second **(B)** stages of the training of the OTSOM model.

As described in Figure 5B, the 2nd stage of training commences with the tonotopically organized ω_*ij*_'s and seeks to train $\theta_{ij}^{r}$'s. The frequency and the phase offset of the external input are sampled from the same set *Y*. During the transient phase, $A_{ij}^{r}$ is typically set to about a tenth of ε so that the entrainment to *I*_*e*_(*t*) can occur. From Figure 8C, it can be observed that entrainment is possible even if *A*_*r*_ is comparable with ε. It can be assumed that the winner oscillator will be the one that is going to satisfy the condition $\left(|{\bar{F}}_{ij}^{net}|\right)$, where ${\overset{\bar{\;}}{F}}_{ij}^{net}$ is the resultant input to the oscillator at (*i, j*) in CAO. $\left|{\bar{F}}_{ij}^{net}\right|$ is a result of constructive or destructive interference between the external input and the input from the SRO, depending on the relative values of ξ_0_ and θ_*r*_, a qualitative explanation of which is discussed in the methods section with Figure 10, and the analytical expression of which is presented by Equations A4.3 and A4.4 in Appendix 4.

The duration of the transient phase (*T*_*sθ*_) in the 2nd stage is typically the same as the duration of the transient phase (*T*_*sω*_) of the 1st stage of training. With the proper choice of the network parameters, principally ε,$A_{ij}^{r}$, μ, μ_*r*_, β_1_, β_1*r*_, as given in Table 1A, the duration of the transient period (*T*_*sθ*_) turned out to be ~3 s. The CAO oscillator whose parameters $\left(\omega_{ij},\theta_{ij}^{r}\right)$ are closest to the input signal parameters (ω_0*p*_, ξ_0*p*_) will be the winner.

In the following Hebbian learning phase, the neighborhood size (*d*_*rθ*_ × *d*_*cθ*_) is dependent on the size of the entrainment window as the Hopf oscillator can only have a stable phase offset when it operates inside its entrainment regime. From Figure 8D, it can be observed that, when the angle of the complex power coupling coefficient ($\theta_{ij}^{r}$) is trained according to the complex Hebbian learning rule (Equation 17) under the 2nd condition (Equation 18b) and Ȧ = 0, it learns the phase offset of the CAO oscillator. The transient response of the CAO oscillator of a single unit in terms of its magnitude of oscillation (*r*), phase offset (δ), entrainment (ω^*^) during the transient phase is plotted in Figure 7C, where the analytically derived solutions of *r* and δ (refer to Appendix 4) are verified numerically. Similarly, the dynamic response of the same CAO oscillator along with the angle of the complex modified power coupling coefficient in the given single unit in terms of *r*, δ, ω^*^, and θ_*r*_ during the Hebbian plasticity phase is presented in Figure 8D, where the analytically derived solutions of *r*, δ, and ω (refer to Appendix 3) are verified numerically. As ω_*ij*_ parameters are self-organized in a monotonically increasing fashion along the x-axis and remain almost invariant along the other orthogonal dimension, the span of the trainable neighborhood window along the x-axis has to be chosen dependent on the entrainment width of the cortical oscillators.

**Figure 11:**
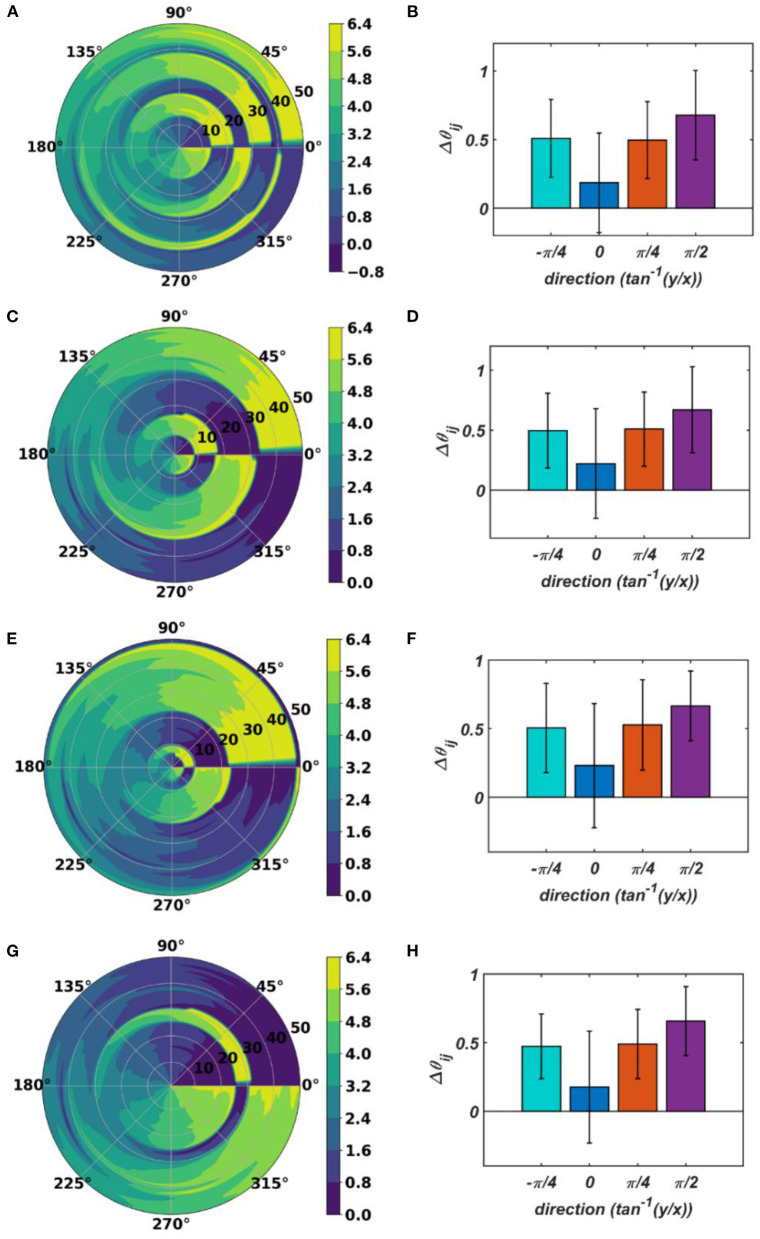
The self-organized $\theta_{ij}^{r}$ parameters after the 2^nd^ stage of the training on four separate instances starting with different randomly initialized $\theta_{ij}^{r}$'s. The subplots **(B)**, **(D)**, **(F)**, and **(H)** represent the mean and the standard deviation of the gradients along the radial (0), azimuth ($\frac{\pi }{2}$), spirally diverging ($\frac{\pi }{4}$) and converging ($-\frac{\pi }{4}$) direction on a polar coordinate representation of the self-organized $\theta_{ij}^{r}$s presented in the subplots **(A)**, **(C)**, **(E)**, and **(G)**, respectively, after the 2nd stage of the training.

With the parameters in Table 1D, from Figure 11, it can be observed that the $\theta_{ij}^{r}$ parameters have self-organized with a linear gradient along the y-axis but almost invariant along the x-axis. Figure 11 represents four different instances of 2nd stage of training with the pre-trained ω_*ij*_ parameters after the 1st stage of the training as presented in Figure 9. The common features about all these four instances are: $\theta_{ij}^{r}$s have either self-organized themselves in a linearly increasing or a decreasing fashion along the y-axis or the azimuth direction in the polar coordinate system representation; it is unpredictable where exactly along the x-axis the self-organized $\theta_{ij}^{r}$s switch from an increasing to decreasing fashion and *vice-versa*, the overall gradients of the self-organized $\theta_{ij}^{r}$s along the 4 axes (x, y and the axes inclined at an angle $\frac{\pi }{4}$ and $-\frac{\pi }{4}$ w.r.t, the x-axis) for all these four instances are statistically similar. If the Gaussian shape of the neighborhood function of ω_*ij*_, ${\eta_{\omega }}_{ij}^{mn}$, defined by its standard deviations along x- and y-axis (σ_*xω*_, σ_*yω*_), is compared with the Gaussian shape of the neighborhood function of $\theta_{ij}^{r}$, ${\eta_{\theta }}_{ij}^{mn}$ (σ_*xθ*_, σ_*yθ*_), the variances of ${\eta_{\theta }}_{ij}^{mn}$ are much smaller than the variances of ${\eta_{\omega }}_{ij}^{mn}$, particularly along the y-axis. Also, the distribution of ${\eta_{\theta }}_{ij}^{mn}$ has a similar spread along both of the orthogonal axes. Distribution of ω_*ij*_s from Figure 9D compared to the distributions of $\theta_{ij}^{r}$s presented in Figures 11B,D–F confirms that they self-organize along orthogonal directions.

### Testing

During the testing phase of the OTSOM model, the conventional power coupling is used from the SRO to the CAO oscillator. The dynamics of the model during the testing phase is described as below:


(19a) $$\begin{array}{l} \dot{r_{ij}}\;=\;\left(\mu -\beta_{1}{r_{ij}}^{2}\right)r_{ij}+A_{ij}^{r}{r_{r}}^{\frac{\omega_{ij}}{\omega_{r}}}\cos\omega_{ij}\left(\frac{\theta_{ij}^{r}}{\omega_{ij}}+\frac{\emptyset_{r}}{\omega_{r}}\;-\;\frac{\emptyset_{ij}}{\omega_{ij}}\right)\\ +\varepsilon \;real\left(I\left(t\right)e^{\;-\;\emptyset_{ij}}\right) \end{array}$$

(19b) $$\begin{array}{l} \dot{\emptyset_{ij}}=\omega_{ij}+A_{ij}^{r}\frac{{r_{r}}^{\frac{\omega_{ij}}{\omega_{r}}}}{r_{ij}}\sin\omega_{ij}\left(\frac{\theta_{ij}^{r}}{\omega_{ij}}+\frac{\emptyset_{r}}{\omega_{r}}\;-\;\frac{\emptyset_{ij}}{\omega_{ij}}\right)\\ +\;\frac{\varepsilon }{r_{ij}}\;imag\left(I\left(t\right)e^{\;-\;\emptyset_{ij}}\right) \end{array}$$

(19c) $$\begin{array}{l} \dot{r_{r}}=\left(\mu_{r}\;-\;\beta_{1r}{r_{r}}^{2}\right)r_{r} \end{array}$$

(19d) $$\begin{array}{l} \dot{\emptyset_{r}}\;=\;\omega_{r} \end{array}$$

**Figure 12:**
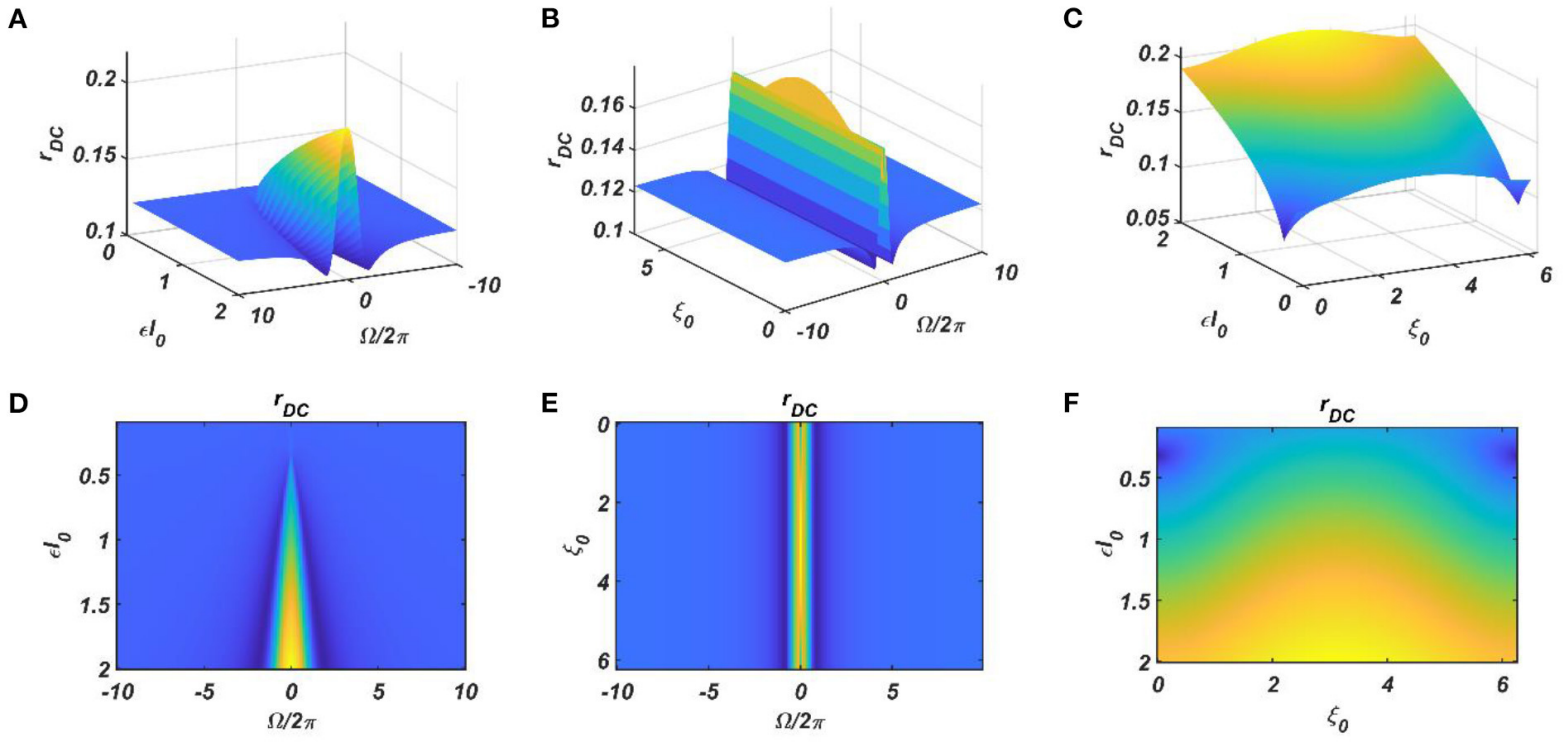
The DC response of the CAO oscillator w.r.t the three properties of the external input signal: amplitude (ε*I*_0_), frequency (ω_0_), and phase offset (ξ_0_). For all these simulations, the common parameters are: μ = 1, β_1_ = −150, ω = 2π × 60, *F* = 1, μ_*r*_ = 1, β_1*r*_ = −10, ω_*r*_ = 2π × 60, *A*_*r*_ = 0.5, θ_*r*_ = π. For the plots, ω_0_ is varied from 2π × 50 *to* 2π × 70, ξ_0_ is varied from 0 to 2π, ε is varied from 0.1 to 2. For the plots in the left column **(A,D)**, ξ_0_ is kept fixed at a value of π. For the plots in the middle column **(B,E)**, ε is kept fixed at a value of 1. For the plots in the left column **(C,F)**, ω_0_ is kept fixed at a value of 2π × 60.

**Figure 13:**
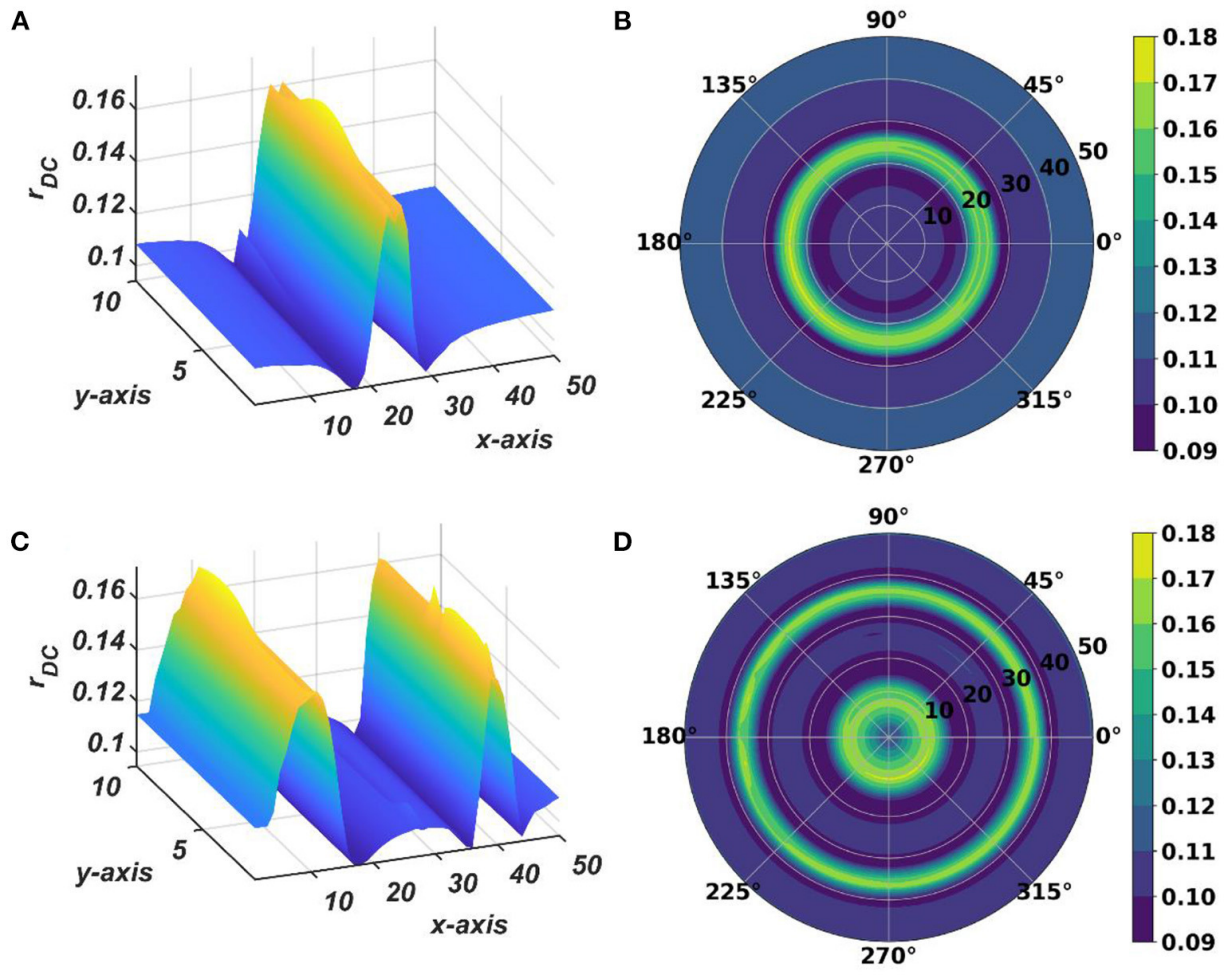
The subplots **(A,B)** depict the response of the OTSOM model when the external input signal is: *I*(*t*) = cos(2π × 57.7029+π). The subplots **(C,D)** depict the response of the OTSOM model when the external input signal is: $I\left(t\right)=\cos\left(2\pi \times 61.426+\frac{3\pi }{2}\right)+\cos\left(2\pi \times 55.669+\frac{\pi }{2}\right)$. The $\omega_{ij}^{\prime }s$ of the CAO oscillators and the $\theta_{ij}^{r}$'s are adopted from the training stage of the OTSOM model as depicted in Figures 9, 11C. The other parameters of the model are mostly preserved as used in the training session of the model: μ = 1, β_1_ = 150, *F* = 1, μ_*r*_ = 1, β_1*r*_ = 1, ω_*r*_ = 2π × 60, *A*_*r*_ = 0.1.

It can be observed that the ratio of the natural frequencies of the CAO oscillators w.r.t, the reference oscillator, is raised to power of the complex activation of the reference oscillator instead of the actual frequencies of the CAO oscillator w.r.t, the reference oscillator. For the moment, real sinusoidal signals or linear combination of multiple real sinusoidal signals is used as external input (*I*(*t*)). At first, we are going to analyze the steady-state response of a single CAO oscillator by considering the DC component of the magnitude of oscillation of the CAO oscillator numerically. The DC component of the steady-state magnitude of oscillation is extracted using a third-order low pass filter with a cut-off frequency of .01 Hz. From Figure 12, it can be observed that the DC component of the steady-state magnitude of oscillation preserves the encoding ability of all the characteristic components of the sinusoidal input signal. The resonance exhibited w.r.t, the frequency of real sinusoidal external input, is similar to the case of complex sinusoidal external input as observed in Figures 13A,D. Whereas the resonance exhibited w.r.t, the phase offset of real sinusoidal external input, is confined to a very narrow bandwidth of the frequency of real sinusoidal external input w.r.t, the natural frequency of the CAO oscillator (Figures 13B,E).

**Figure 14:**
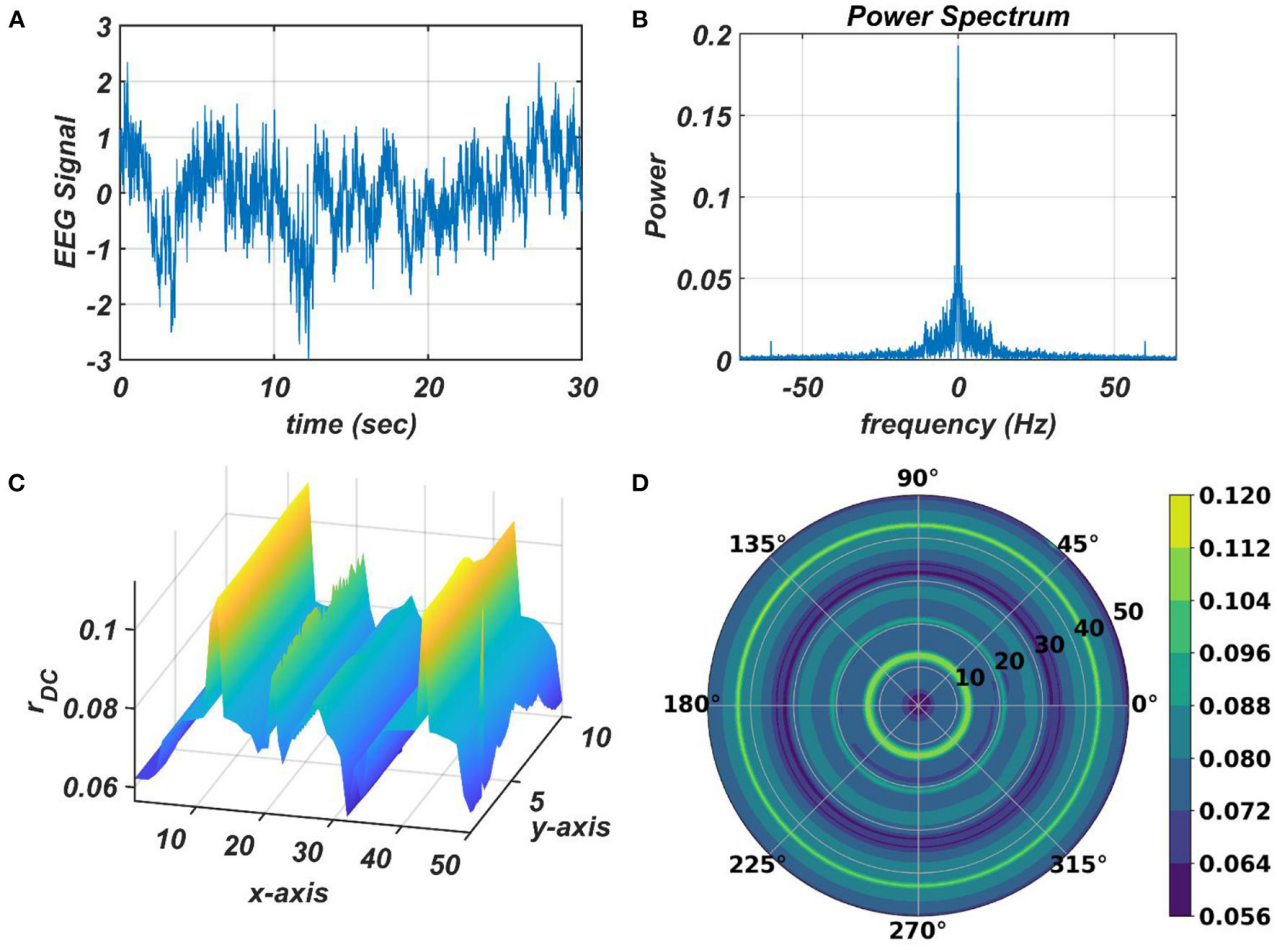
The subplots **(C,D)** illustrate the response of the OTSOM model when an arbitrary aperiodic signal such as the EEG signal plotted in **(A)** characterized by its power spectrum plotted in **(B)** is presented as an external input signal.

The response of the OTSOM model is tested with three types of signals: the periodic real sinusoidal signal (Figures 13A,B), the quasi-periodic signal, which is a combination of two proximal frequency components (Figures 13C,D), given in the caption of Figure 13 and an aperiodic signal (Figures 14C,D) such as an Electroencephalograph (EEG) signal as plotted in Figure 14A with its characterizing power spectrum plotted in Figure 14B. The EEG signal was collected during a mind-wondering task, with a sampling rate of 1,024 Hz (Grandchamp et al., 2014). The characterizing ω_*ij*_ and $\theta_{ij}^{r}$ parameters during the testing phase are illustrated in Figures 9, 12C, respectively. It can be observed from Figures 13C,D that the representation of the frequency 55.669 Hz is broader w.r.t, the frequency 61.426 Hz, which is because of the slope of ω_*ij*_s along the x-axis at around 55.669 Hz is lesser than the slope at around 61.426 Hz.

## Discussion

The tonotopic map refers to a map of tones or individual frequencies often found in auditory cortices of mammals. Optimal response at a specific frequency is a characteristic of resonance. Based on this insight, we designed a tonotopic map model based on nonlinear oscillators capable of exhibiting resonance. We present a model of a tonotopic map, which consists of an array of Hopf oscillators, labeled as CAO. The map is trained in complex sinusoidal stimuli such that the frequency is mapped onto the columns, and the phase is mapped onto the rows. (The phase of the input signal is defined with reference to a reference oscillator labeled as SRO). In other words, when a complex sinusoid with a given frequency and the phase is presented as an input stimulus, the oscillator at a specific row and a column, whose frequency and phase are the closest to the input parameters, responds with the highest amplitude.

Existing computational models of the tonotopic map do not attempt to model the underlying oscillation or the associated resonance in modeling tuned responses to pure tones. In the tonotopic model of (Ritter et al., 1992), which is based on a SOM model, frequencies are modeled as explicit parameters defined out of the context of the underlying oscillatory process. The model was able to achieve an ordered map of frequencies, with greater areas of the map differentially allotted to dominant frequencies in the input. However, the model was not able to capture any other temporal aspects of the input signal, since no signal was explicitly modeled. Another tonotopic map model described by Palakal et al. (1995) modeled the distribution of both frequency and time delay. But, here, too, these parameters are described as independent parameters, taken out of the context of the underlying temporal process. In this regard, the proposed tonotopic model based on oscillators and resonance represents a significant step forward.

A previous model (Biswas et al., 2021) that shows how a network of Hopf oscillators can be trained to learn arbitrary aperiodic signals was developed further to create the proposed tonotopic map model. To this end, two improvements had to be made to the previous model:

a) a key element of (Biswas et al., 2021) is the concept of power coupling that achieves a stable (normalized) phase relationship between a pair of oscillators with arbitrary intrinsic frequencies. This scheme had to be modified in the proposed model since it must allow mixed forms of coupling, combining power coupling with ordinary real coupling.
b) in the proposed model, oscillators must exhibit tuned responses not only to frequency but also to phases. In order to define a phase offset of the input signal, we introduced a reference oscillator (SRO) that projects to all the oscillators in the map.

The functional unit of the OTSOM model is a single CAO oscillator that receives input from the external input and the SRO. We performed the qualitative and the quantitative analysis of this unit under two conditions:

1. Magnitude of the SRO input is negligible compared to the external input.
2. Magnitude of the SRO input is comparable to the external input.

The magnitude of the input from the reference oscillator is $A_{r}{r_{r}}^{\frac{\omega^{*}}{\omega_{r}^{*}}}$, where the steady-state value of the magnitude of oscillation of the SRO depends on μ_*r*_ and β_1*r*_. As the SRO operates in the supercritical Hopf regime, both μ_*r*_ and β_1*r*_ are positive, and the steady-state magnitude of oscillation is $\sqrt{\frac{\mu_{r}}{\beta_{1r}}}$. With this simple setup, we have observed that the CAO oscillator can encode not only the frequency of the complex sinusoidal input signal primarily inside the entrainment regime but also the phase offset of the input signal.

The entrainment regime of a canonical Hopf oscillator is previously analyzed by (Kim and Large, 2015) in terms of analyzing steady-state dynamical characteristics on the *r* − ψ plane. As ψ is the angular difference between the oscillator and the external input signal, when the system exhibits a stable fixed point (> 0, *T*^2^ − 4 > 0, *T* < 0) or stable spiral behavior (> 0, *T*^2^ − 4 < 0, *T* < 0), it can be interpreted that the system is entrained. When the relative phase of the oscillator w.r.t, the input signal reaches a steady-state value, it essentially means the actual frequency of oscillation of the oscillator is adapted from its natural frequency of oscillation to the frequency of the driving signal. Kim and Large (Kim and Large, 2015) have analyzed the effect of the strength of the driving signal on its entrainment characteristics by mapping the nature of the steady-state solution on the ε*F* vs Ω space. There are five possible steady-state solutions exhibited by four regimes of the canonical Hopf oscillator defined by its intrinsic parameter values. These five steady-state solutions are the stable node, the stable spiral, the unstable node, the unstable spiral, and the saddle point. When the Hopf oscillator operates in critical Hopf parameter regime (μ = 0, β_1_ > 0, β_2_ = 0), it exhibits either the stable node or the stable spiral solution at steady state, i.e., for any values of its intrinsic parameter β_1_, the strength of the driving signal (ε*F*) and Ω, it is going to be entrained to the frequency of the driving signal. Therefore, it can be stated that the entrainment regime of the Hopf oscillator operating in the critical parameter regime is unbounded. In both of these cases, the state of the system reaches the fixed point asymptotically, i.e., it takes forever for the oscillator to get entrained. Generally, a small neighborhood around the fixed point is defined to declare the entrainment of the system. The Hopf oscillator operating in the supercritical parameter regime (μ > 0, β_1_ > 0, β_2_ = 0) exhibits three steady-state solutions: the stable fixed point, the stable spiral, and the unstable spiral. Till the boundary to the unstable spiral solution, the system exhibits entrainment.

The typical initial value of the variance η_ω_ has the property: σ_*yωm*_ ≫ σ_*xωm*_. Due to high variance along the column, the other oscillators in the same column as the winner tend to adapt to the feature of the presented input pattern at the same rate as the winner neuron, which ensures the low variability of the learned natural frequency of the oscillator along *y*- axis. An initial standard deviation of σ_*yωm*_ = 100 and σ_*xωm*_ = 4 is sufficient for the tonotopic organization to arise as presented in Figure 9. Although there is a lower bound for σ_*yωm*_, depending on the number of oscillators along the *y*- direction, *N*_*y*_, there is no strict bound on σ_*xωm*_ depending on the dimensionality of the 2D array of oscillators. It can be interpreted that the lower bound on σ_*yωm*_ should be proportional to *N*_*y*_, as the greater the number of oscillators per column, the lesser the adaptability rate of the oscillators at the boundaries of the adaptable neighborhood along the column. However, a square neighborhood window function is chosen for the simulation presented in this study; a rectangular window function is also feasible. A rectangular window function can be defined by *d*_*r*_ ≠ *d*_*c*_. σ_*xω*_ and σ_*yω*_ decrease in a Gaussian contour w.r.t time at a much slower time scale to model the effect of annealing. σ_*xω*_ and σ_*yω*_ are updated after every epoch, with a typical standard deviation on an iterative time scale of 500ñ_*eph*, ω_.

A few aspects need to be elaborated about the 2^nd^ stage of the training. The $\theta_{ij}^{r}$s were failing to self-organize themselves in a linearly increasing or decreasing fashion along the column when the CAO oscillators were placed on a 2-dimensional rectangular grid. To fix this issue, periodic boundary condition is introduced along the spatial dimension of the column, i.e., the bottom row of the CAO is closest with an equidistant to both the top row as well as the second last row, which is the motivation behind representing the CAO on a polar coordinate representation. Although this ensured that the $\theta_{ij}^{r}$s self-organize themselves in a linearly increasing or decreasing manner in a given column, $\theta_{ij}^{r}$s were slipping at a constant rate along the azimuth axis, which can be observed in Supplementary Figure S1A. To fix this problem, the $\theta_{ij}^{r}$s of the top most row of the CAO were fixed at 0^*o*^ angle, and, from Supplementary Figure S1B, it can be observed that $\theta_{ij}^{r}$s of a given column were stabilized with a linear organization. On the contrary, the ω_*ij*_s are able to self-organize themselves without the aforementioned periodic boundary condition.

A comparison with the conventional SOM: For the conventional SOM model (Kohonen, 1998), the neuronal response is characterized by its linear or nonlinear activation function. When these rate-coded neurons are a part of the SOM framework, the afferent weights for a particular neuron are also considered to be an internal feature of the neurons, considering close proximity of these afferent synapses to the corresponding neurons. The key differences between the conventional SOM model and OTSOM are:

1. The afferent connection weights are fixed.
2. The input is a time-varying complex sinusoidal signal instead of a constant vector.
3. The neurons are limit-cycle oscillators instead of rate-coded neurons with a static transfer function.

Although we have tested the model with complex sinusoidal input signals sampled from the frequency band from 55 to 65 Hz, the bandwidth can be scaled up/down or shifted. The proposed model can be used to explain the tonotopic organization evolved in the auditory cortex of mammals.

## Data availability statement

The original contributions presented in the study are included in the article/Supplementary material, further inquiries can be directed to the corresponding author/.

## Author contributions

DB and AT: hypothesis testing, conceptualization, theory development, numerical simulations, investigation, methodology, and validation. DB and VC: visualization and writing—original draft. VC: writing—review, editing, and supervision. All the authors contributed to the article and approved the submitted version.

## Conflict of interest

The authors declare that the research was conducted in the absence of any commercial or financial relationships that could be construed as a potential conflict of interest.

## Publisher's note

All claims expressed in this article are solely those of the authors and do not necessarily represent those of their affiliated organizations, or those of the publisher, the editors and the reviewers. Any product that may be evaluated in this article, or claim that may be made by its manufacturer, is not guaranteed or endorsed by the publisher.

## References

[B1] Aronson D. G. Ermentrout G. B. Kopell N. (1990). Amplitude response of coupled oscillators. Phys. D. 41, 403–449.

[B2] Bates M. E. Simmons J. A. Zorikov T. V. (2011). Bats use echo harmonic structure to distinguish their targets from background clutter. Science 333, 627–630. 10.1126/science.1202065 21798949

[B3] Biswas D. Pallikkulath S. Chakravarthy V. S. (2021). A complex-valued oscillatory neural network for storage and retrieval of multidimensional aperiodic signals. Front. Comput. Neurosci. 15, 1–24. 10.3389/fncom.2021.551111 34108869 PMC8181409

[B4] Boynton G. M. Stecker G. C. Huber E. Thomas J. M. Saenz M. Fine I. (2015). Population receptive field estimates of human auditory cortex. NeuroImage 206, 428–439. 10.1016/j.neuroimage.2014.10.060 25449742 PMC4262557

[B5] Clopton B. M. Winfield J. A. Flammino F. J. (1974). Tonotopic organization: review and analysis. Brain Res. 76, 1–20. 4367399 10.1016/0006-8993(74)90509-5

[B6] Eguíluz V. M. Ospeck M. Choe Y. Hudspeth A. J. Magnasco M. O. (2000). Essential nonlinearities in hearing. Phys. Rev. Lett. 84, 20–23. 10.1103/PhysRevLett.84.5232 10990910

[B7] Ehret G. Romand R. (1996). The Central Auditory System, eds Ehret G. Romand R. (Oxford: Oxford University Press).

[B8] Farokhniaee A. Almonte F. V. Yelin S. Large E. W. (2020). Entrainment of weakly coupled canonical oscillators with applications in gradient frequency neural networks using approximating analytical methods. Mathematics. 8, 1312. 10.3390/math8081312

[B9] Frank J. Andor D. Duke T. (2001). Physical basis of two-tone interference in hearing. PNAS 98, 9080–9085. 10.1073/pnas.151257898 11481473 PMC55376

[B10] Fredrickson-hemsing L. Strimbu C. E. Roongthumskul Y. Bozovic D. (2012). Dynamics of freely oscillating and coupled hair cell bundles under mechanical deflection. Biophys. J. 102, 1785–1792. 10.1016/j.bpj.2012.03.017 22768934 PMC3328720

[B11] Grandchamp R. Braboszcz C. Delorme A. (2014). Oculometric variations during mind wandering. Front. Psychol. 5, 1–10. 10.3389/fpsyg.2014.00031 24575056 PMC3920102

[B12] Hadjikhani N. Liu A. K. Dale A. M. Cavanagh P. Tootell R. B. H. (1998). Retinotopy and color sensitivity in human visual cortical area V8. Nat. Neurosci. 1, 235–241. 10195149 10.1038/681

[B13] Hoppensteadt F. C. Izhikevich E. M. (1996). Synaptic organizations and dynamical properties of weakly connected neural oscillators I. Analysis of a canonical model. Biol. Cybern. 127, 117–127. 8855350 10.1007/s004220050279

[B14] Hoppensteadt F. C. Izhikevich E. M. (1997). Weakly Connected Neural Networks, Vol. 126. New York, NY: Springer. 10.1007/978-1-4612-1828-9

[B15] Hubel D. H. Wiesel T. N. (1959). Receptive fields of single neurones in the cat's striate cortex. J. Physiol. 148, 574–591. 14403679 10.1113/jphysiol.1959.sp006308 PMC1363130

[B16] Imig T. J. Adrian H. O. (1977). Binaural columns in the primary field (A1) of cat auditory cortex. Brain Res. 138, 241–257. 589474 10.1016/0006-8993(77)90743-0

[B17] Kern A. Stoop R. (2003). Essential role of couplings between hearing nonlinearities. Phys. Rev. Lett. 91, 3–6. 10.1103/PhysRevLett.91.128101 14525401

[B18] Kim J. C. Large E. W. (2015). Signal processing in periodically forced gradient frequency neural networks. Front. Comput. Neurosci. 9, 1–14. 10.3389/fncom.2015.00152 26733858 PMC4689852

[B19] Kim J. C. Large E. W. (2019). Mode locking in periodically forced gradient frequency neural networks. Phys. Rev. E 99, 022421. 10.1103/PhysRevE.99.022421 30934299

[B20] Kim J. C. Large E. W. (2021). Multifrequency Hebbian plasticity in coupled neural oscillators. Biol. Cybern. 115, 43–57. 10.1007/s00422-020-00854-6 33399947

[B21] Kohonen T. (1988). An introduction to neural computing. Neural Netw. 1, 3–16.

[B22] Kohonen T. (1990). The self-organizing map. Proc. IEEE 78, 1464–1480.

[B23] Kohonen T. (1998). The self-organizing map. Neurocomputing. 21, 1–6.

[B24] Large E. W. Almonte F. V. Velasco M. J. (2010). A canonical model for gradient frequency neural networks. Phys. D. 239, 905–911. 10.1016/j.physd.2009.11.015

[B25] Lerud K. D. Kim J. C. Almonte F. V. Carney L. H. Large E. W. (2019). A canonical oscillator model of cochlear dynamics. HHS Public Access. 380, 100–107. 10.1016/j.heares.2019.06.001 31234108 PMC6669083

[B26] Merzenich M. M. Kaas J. H. Roth G. L. (1976). Auditory cortex in the grey squirrel: tonotopic organization and architectonic fields. J. Comp. Neurol. 166, 387–401. 1270613 10.1002/cne.901660402

[B27] Merzenich M. M. Knight P. L. Roth G. L. (2018). Representation of cochlea within primary auditory cortex in the cat. J. Neurophysiol. 38, 231–249. 10.1152/jn.1975.38.2.231 1092814

[B28] Novick A. Vaisnys J. R. (1964). Echolocation of flying insects by the bat, Chilonycteris parnellii. Biol. Bull. 127, 478–488.

[B29] Palakal M. J. Murthy U. Chittajallu S. K. Wong D. (1995). Tonotopic representation of auditory responses using self-organizing maps. Math. Comput. Model. 22, 7–21.

[B30] Palmer A. R. Rees A. (2010). The Oxford Handbook of Auditory Science: The Auditory Brain, eds Palmer A. R. Rees A. (Oxford: Oxford University Press). 10.1093/oxfordhb/9780199233281.001.0001

[B31] Penfield B. Y. W. (1937). Somatic motor and sensory representation in. Brain. 60:389–443.

[B32] Righetti L. Buchli J. Ijspeert A. J. (2005). From dynamic hebbian learning for oscillators to adaptive central pattern generators, in Proceedings of 3rd International Symposium on Adaptive Motion in Animals and Machines AMAM.p. 1–7.

[B33] Ritter H. Martinez T. Schulten K. (1992). Neural Computation and Self-Organizing Maps: An Introduction. Boston: Addison-Wesley.

[B34] Ruggero M. A. (1992). Responses to sound of the basilar membrane of the mammalian cochlea. Curr. Opin. Neurobiol. 2, 449–456. 1525542 10.1016/0959-4388(92)90179-o PMC3579517

[B35] Schreiner C. E. Sutter L. (1992). Topography of excitatory bandwidth in cat primary auditory cortex: single-neuron versus multiple-neuron recordings. J. Neurophysiol. 68, 1487–1502. 1479426 10.1152/jn.1992.68.5.1487

[B36] Simmons J. A. (2012). Bats use a neuronally implemented computational acoustic model to form sonar images. Curr. Opin. Neurobiol. 22, 311–319. 10.1016/j.conb.2012.02.007 22436892

[B37] Strogatz S. H. (1994). Nonlinear Dynamics and Chaos. Boston: Addison Wesley.

[B38] Suga N. (1990). Biosonar and neural computation in bats. Sci. Am. 262, 60–68. 2343295 10.1038/scientificamerican0690-60

[B39] Suga N. Yan J. Zhang Y. (1997). Cortical maps for hearing and egocentric selection for self-organization. Trends Cognit. Sci. 1, 13–20. 21223848 10.1016/S1364-6613(97)01002-4

[B40] von Bekesy G. (1949). The vibration of the cochlear partition in anatomical preparations and in models of the inner ear. J. Acoust. Soc. Am. 240, 233–245.

[B41] Wandell B. A. Dumoulin S. O. Brewer A. A. (2007). Review visual field maps in human cortex. Neuron 1893, 366–383. 10.1016/j.neuron.2007.10.012 17964252

[B42] Yue X. Robert S. Ungerleider L. G. (2020). Curvature processing in human visual cortical areas. NeuroImage 222, 117295. 10.1016/j.neuroimage.2020.117295 32835823 PMC7885662

