# Supplementary material for: Modeling the tonotopic map using a two-dimensional array of neural oscillators
Source: Front Comput Neurosci. 2022 Aug 24;16:909058. doi: 10.3389/fncom.2022.909058 (PMC9450043; doi:10.3389/fncom.2022.909058)
Supplement: Supplementary file 1 [file Data_Sheet_1.docx]

Supplementary Material

# Appendix

**Appendix 1: A pair of Hopf oscillators coupled bilaterally and unilaterally through modifier power coupling.**

The dynamics of a pair of Hopf oscillators bilaterally coupled through modified power coupling is described by the eqns. 6,7 and 8. Whereas the dynamics of a pair of Hopf oscillators unilaterally coupled through modified power coupling which is identical as the single unit of the OTSOM model under the special condition $\varepsilon\cong0$ is described by eqns. 12, 13 and 14. The schematic of these networks are shown in the fig. A1.


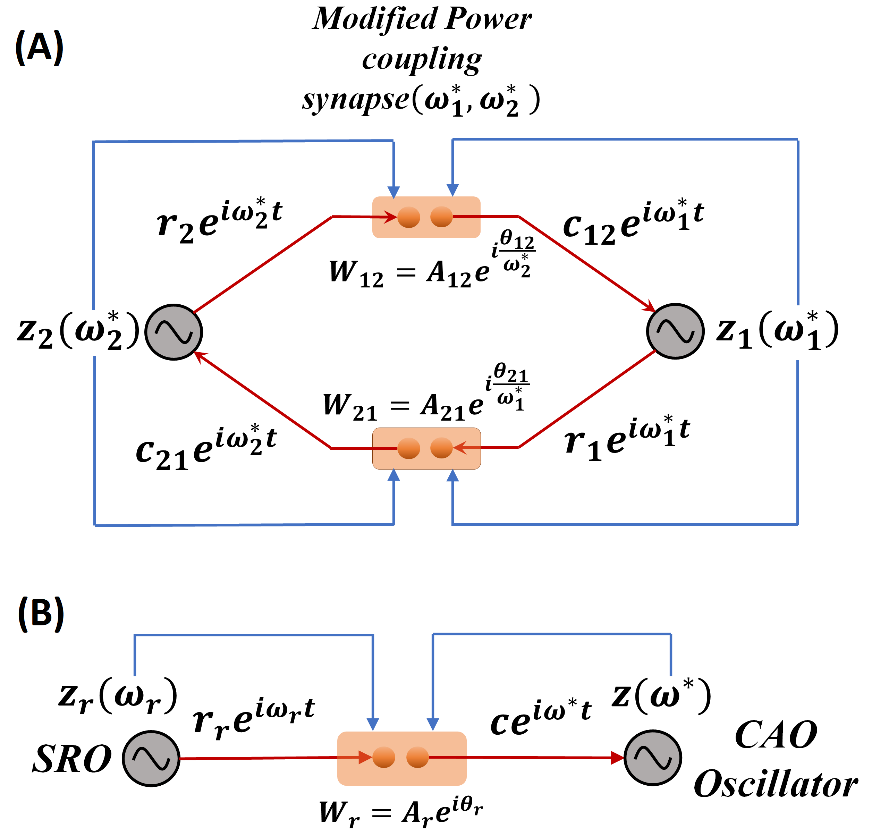


**Figure A1:** Network schematic of a pair of Hopf oscillators bilaterally **(A)** and unilaterally **(B)** coupled through modified power coupling.

**Network 1 (bilateral coupling):** Initially both the oscillators will receive complex sinusoidal input signal from the other oscillator through modified power coupling connection as $\omega_{1}^{*}$ and $\omega_{2}^{*}$ are initialised to $\omega_{1}$ and $\omega_{2}$ respectively. As the Hopf oscillator keeps oscillating at its natural frequency, $\omega$, when it is perturbed by a complex sinusoidal signal with the same frequency, $\omega_{0}=\omega$, both of the oscillators in the pair will continue to oscillate at a same frequency. So, $\omega_{1}^{*}$ and $\omega_{2}^{*}$ will remain as $\omega_{1}$ and $\omega_{2}$. Defining the new normalized phase difference as, $\lambda_{21}=\frac{\emptyset_{1}}{\omega_{1}^{*}}-\frac{\emptyset_{2}}{\omega_{2}^{*}}$, assuming at steady state $\dot{\lambda_{21}}=\frac{\dot{\emptyset_{1}}}{\omega_{1}}-\frac{\dot{\emptyset_{2}}}{\omega_{2}}=0$, considering $\omega_{1}^{*}=\omega_{1}$ and $\omega_{2}^{*}=\omega_{2}$ all along. From equation 2b and 3b,

$$1+\frac{A_{12}{r_{2}}^{\frac{\omega_{1}^{*}}{\omega_{2}^{*}}}}{\omega_{1}r_{1}}\sin\omega_{1}\left( \frac{\theta_{12}}{\omega_{1}\omega_{2}}+\frac{\emptyset_{2}}{\omega_{2}}-\frac{\emptyset_{1}}{\omega_{1}} \right)-1-\frac{A_{21}{r_{1}}^{\frac{\omega_{2}^{*}}{\omega_{1}^{*}}}}{\omega_{2}r_{2}}\sin\omega_{2}\left( \frac{\theta_{21}}{\omega_{1}\omega_{2}}+\frac{\emptyset_{1}}{\omega_{1}}-\frac{\emptyset_{2}}{\omega_{2}} \right)=0$$

$$\frac{A_{12}{r_{2}}^{\frac{\omega_{1}}{\omega_{2}}}}{\omega_{1}r_{1}}\sin\omega_{1}\left( \frac{\theta}{\omega_{1}\omega_{2}}+\frac{\emptyset_{2}}{\omega_{2}}-\frac{\emptyset_{1}}{\omega_{1}} \right)+\frac{A_{21}{r_{1}}^{\frac{\omega_{2}}{\omega_{1}}}}{\omega_{2}r_{2}}\sin\omega_{2}\left( \frac{\theta}{\omega_{1}\omega_{2}}+\frac{\emptyset_{2}}{\omega_{2}}-\frac{\emptyset_{1}}{\omega_{1}} \right)=0$$

As we have already assumed, $\theta_{12}=-\theta_{21}=\theta$, it is obvious that for some of the solutions of the above equation both the terms in the L.H.S of the equation would be 0, in that case;

$$\frac{\theta}{\omega_{1}\omega_{2}}+\frac{\emptyset_{2}}{\omega_{2}}-\frac{\emptyset_{1}}{\omega_{1}}=n\pi$$

$$\lambda_{21}=n_{1}\pi+\frac{\theta}{\omega_{1}\omega_{2}}$$

$$\sigma_{21}=\lambda_{21}-\frac{\theta}{\omega_{1}\omega_{2}}=\frac{\emptyset_{1}}{\omega_{1}}-\frac{\emptyset_{2}}{\omega_{2}}-\frac{\theta}{\omega_{1}\omega_{2}}=n_{1}\pi$$

where, $n_{1}=-n$. So. The normalized phase difference has multiple solutions which depends on $\theta$ as well as the natural frequency of both of the oscillators.

**Network 2 (unilateral coupling):** Initially the CAO oscillators will receive complex sinusoidal input signal from the SRO through modified power coupling connection as $\omega^{*}$ is initialised to $\omega$. As the Hopf oscillator keeps oscillating at its natural frequency, $\omega$, when it is perturbed by a complex sinusoidal signal with the same frequency, $\omega_{0}=\omega$, CAO oscillator will continue to oscillate at a same frequency. So, $\omega^{*}$ will remain as$\omega$. The normalized phase difference between CAO oscillator and SRO will be:

$$\lambda_{r}=\frac{\emptyset}{\omega^{*}}-\frac{\emptyset_{r}}{\omega_{r}}$$

As $\omega^{*}$ will remain $\omega$ the normalised phase difference will be: $\lambda_{r}=\frac{\emptyset}{\omega}-\frac{\emptyset_{r}}{\omega_{r}}$. Assuming $\dot{\lambda_{r}}=0$ at steady-state;

$$\frac{\dot{\emptyset}}{\omega}-\frac{\dot{\emptyset_{r}}}{\omega_{r}}=0$$

$$A_{r}\frac{{r_{r}}^{\frac{\omega}{\omega_{r}}}}{r}\sin\left( \theta_{r}-\lambda_{r}\omega\right)=0$$

The only solution of which is: $\lambda_{r}=\frac{\theta_{r}}{\omega}+n\pi$. So, the pair of unilaterally coupled Hopf oscillators through modified power coupling will always synchronize with each other with a normalized phase difference of $\frac{\theta_{r}}{\omega}+n\pi$.

**Appendix 2: The steady state dynamical analysis of single unit as the CAO oscillator operates under the entrainment regime of the input signal.**

The approximate dynamics of eqns. 12, 13, 14 considering $A_{r}\ll\varepsilon I_{0}$, when $\omega=\omega_{0}$:

$$\dot{r}\approx\left( \mu-\beta_{1}r^{2} \right)r+\varepsilon I_{0}\cos\psi A2.1$$

$$\dot{\psi}\approx-\frac{\varepsilon I_{0}}{r}\sin\psi A2.2$$

$$\tau_{\omega}\dot{\omega^{*}}\approx-\omega^{*}+\omega-\frac{\varepsilon I_{0}}{r}\sin\psi A2.3$$

At steady state, $\dot{r}=0$, $\dot{\psi}=0$. From equation A2.2:

$$\dot{\psi_{ss}}\approx-\frac{\varepsilon I_{0}}{r_{ss}}\sin\psi_{ss}=0$$

$$i.e, \sin\psi_{ss}=0$$

$$i.e, \psi_{ss}^{*}=n\pi$$

The solutions $\psi_{ss}^{*}=\left( 2n+1 \right)\pi$ are unstable whereas the solutions $\psi_{ss}^{*}=2n\pi$ are stable. For the stable solutions;

$$\emptyset_{ss}-\omega_{0}t-\xi_{0}=2\pi n$$

$$\delta_{ss}=2\pi n+\xi_{0}$$

i.e., the steady state phase offset of the CAO oscillator will be same as the phase offset of the input signal. For $\psi_{ss}^{*}=2n\pi$ solutions, equation A2.1 will become:

$$\left( \mu-\beta_{1}{r_{ss}}^{2} \right)r_{ss}+\varepsilon I_{0}\approx0 \left( A2.4 \right)$$

From the last expression, the positive real solution, $r_{ss}$, of (A2.4) is derived with the help of MathWorks equation solver:

$$r_{ss}^{*}=\frac{\sqrt[3]{\frac{2}{3}}\mu}{\sqrt[3]{\sqrt{3}\sqrt{27\varepsilon^{2}{I_{0}}^{2}{\beta_{1}}^{4}+4{\beta_{1}}^{3}\mu^{3}}+9\varepsilon I_{0}{\beta_{1}}^{2}}}-\frac{\sqrt[3]{\sqrt{3}\sqrt{27\varepsilon^{2}{I_{0}}^{2}{\beta_{1}}^{4}+4{\beta_{1}}^{3}\mu^{3}}+9\varepsilon I_{0}{\beta_{1}}^{2}}}{\sqrt[3]{2}3^{\frac{2}{3}}\beta_{1}} \left( A2.5 \right)$$

The solution of steady state magnitude of oscillation as found in eqn. A2.5 is numerically verified in fig. A2.1. Whereas the fig. A2.2 elaborates the dependency of the entrainment width and the typical transient time on the parameters $\mu$, $\beta_{1}$ and $\varepsilon I_{0}$.


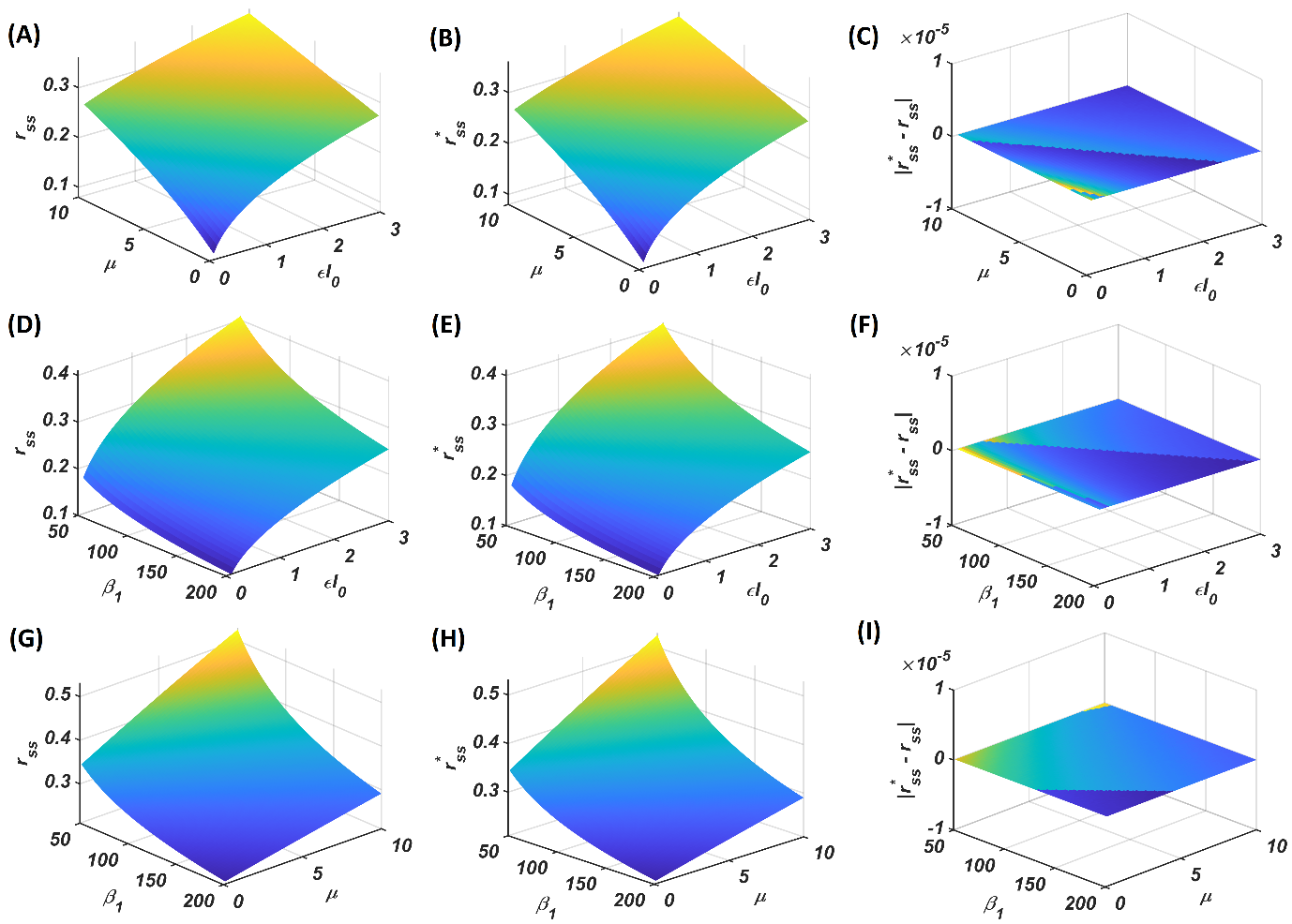


**Figure A2.1:** The first two columns (subplots **A, D, G** and **B, E, H**) proclaim that the dependency of the solution $r_{ss}^{*}$ of the polynomial expression given by eqn.- A2.4 found by MathWorks polynomial solver, given by the expression in eqn.- A2.5 on two of the three parameters $\mu, \beta_{1}$ and $\varepsilon I_{0}$ keeping the remaining fixed at a certain intermediate value ($\varepsilon I_{0}=2, \beta_{1}=150, \mu=1$) is identical to the steady state magnitude of oscillation ($r_{ss}$) deduced by simulating the transient dynamics of eqns.- A2.1 and A2.2 with $\omega=\omega_{0}=2\pi\times60$. The error between the first two columns is plotted in the 3^rd^ column (subplots **C, F, I**).


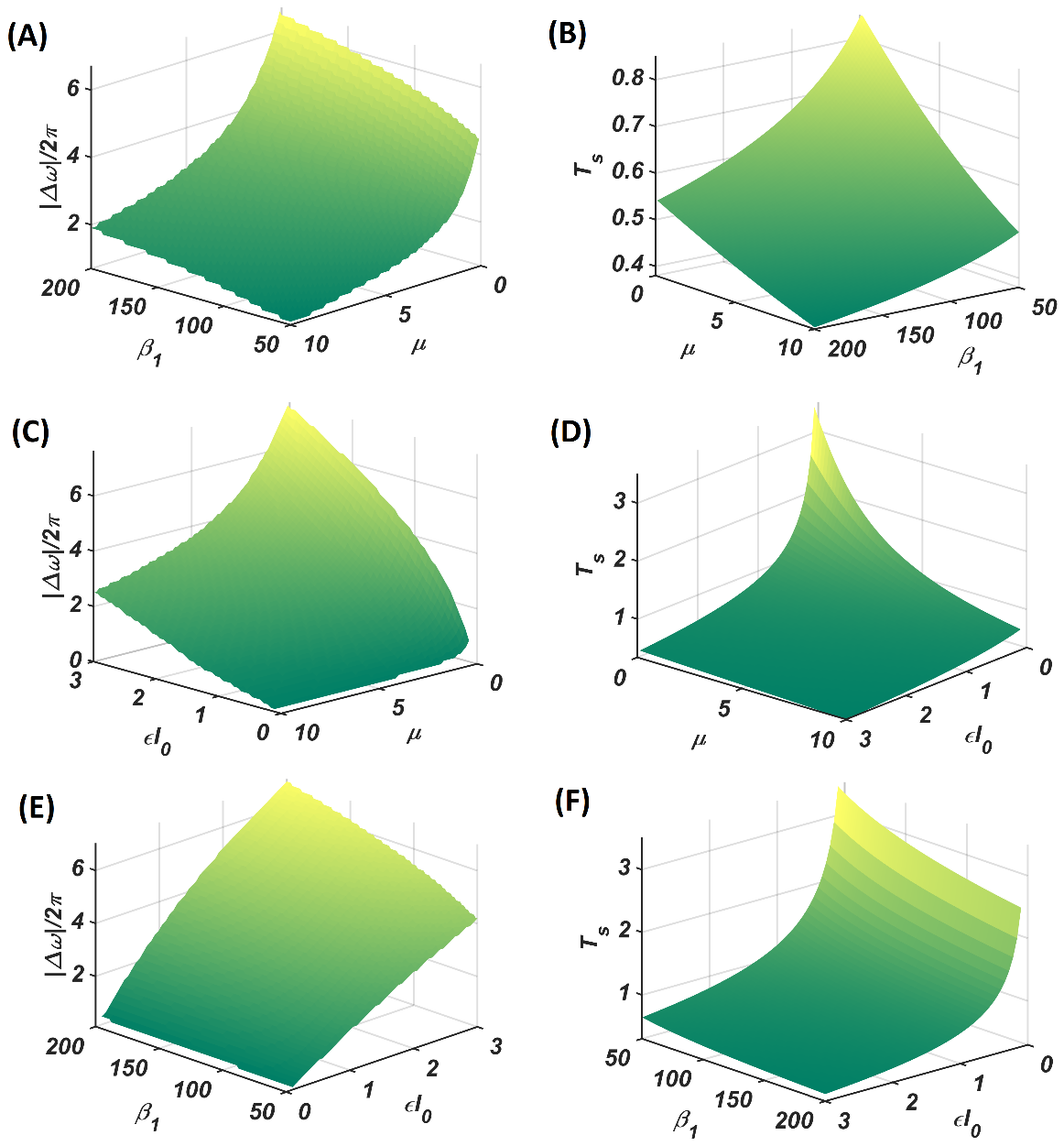


**Figure A2.2:** The subplots **A**, **C**, **E** delineates the dependency of the width of the entrainment regime ($\Delta\omega$) on two of the three parameters $\mu, \beta_{1}$ and $\varepsilon$ keeping the remaining fixed at a certain intermediate value, similarly the remaining subplots delineates the dependency of $T_{s}$ (the time an individual oscillator takes to attain steady-state under the influence of complex sinusoidal external input signal). Parameter value: For **(A)** and **(B)** $\varepsilon I_{0}=2$, for **(C)** and **(D)** $\beta_{1}=150$ and for **(E)** and **(F)** $\mu=1$, $\omega$ is kept fixed at $2\pi\times60$ rad/sec while $\omega_{0}$ is varied from $2\pi\times45$ rad/sec to $2\pi\times75$ rad/sec.

The approximate dynamics when the CAO oscillator is under the entrainment regime with $\omega\neq\omega_{0}$:

$$\dot{r}\approx\left( \mu-\beta_{1}r^{2} \right)r+\varepsilon I_{0}\cos\psi A2.6$$

$$\dot{\psi}\approxΩ-\frac{\varepsilon I_{0}}{r}\sin\psi A2.7$$

At steady state, equating eqn. A2.7 to zero;

$$Ω-\frac{\varepsilon I_{0}}{r_{ss}}\sin\psi_{ss}=0$$

$$\sin\psi_{ss}=\frac{Ωr_{ss}}{\varepsilon I_{0}}$$

Substituting $\cos\psi_{ss}$ into eqn. A2.4 with the steady state assumption,

$$\left( \mu-\beta_{1}{r_{ss}}^{2} \right)r_{ss}+\varepsilon I_{0}\sqrt{1-\left( \frac{Ωr_{ss}}{\varepsilon I_{0}} \right)^{2}}=0$$

$${\beta_{1}}^{2}{r_{ss}}^{6}-2\mu\beta_{1}{r_{ss}}^{4}+\left( \mu^{2}+Ω^{2} \right){r_{ss}}^{2}-\varepsilon^{2}{I_{0}}^{2}=0$$

The solution of the expression can be drawn using MathWorks polynomial solver. The only positive real solution of the equation is:

$$r_{ss}^{*}=\left( \frac{1}{3\sqrt[3]{2}\beta_{1}^{2}}\sqrt[3]{27\beta_{1}^{4}\varepsilon^{2}{I_{0}}^{2}+18\beta_{1}^{3}\Omega^{2}\mu+2\beta_{1}^{3}\mu^{3}+\sqrt{4\left( 3\beta_{1}^{2}\Omega^{2}-\beta_{1}^{2}\mu^{2} \right)^{3}+\left( 27\beta_{1}^{4}\varepsilon^{2}{I_{0}}^{2}+18\beta_{1}^{3}\Omega^{2}\mu+2\beta_{1}^{3}\mu^{3} \right)^{2}}}-\frac{\sqrt[3]{2}\left( 3\beta_{1}^{2}\Omega^{2}-\beta_{1}^{2}\mu^{2} \right)}{3\beta_{1}^{2}\sqrt[3]{27\beta_{1}^{4}\varepsilon^{2}{I_{0}}^{2}+18\beta_{1}^{3}\Omega^{2}\mu+2\beta_{1}^{3}\mu^{3}+\sqrt{4\left( 3\beta_{1}^{2}\Omega^{2}-\beta_{1}^{2}\mu^{2} \right)^{3}+\left( 27\beta_{1}^{4}\varepsilon^{2}{I_{0}}^{2}+18\beta_{1}^{3}\Omega^{2}\mu+2\beta_{1}^{3}\mu^{3} \right)^{2}}}}-\frac{2\mu}{3\beta_{1}} \right)^{\frac{1}{2}}$$

$$r_{ss}^{*}=\left( \frac{\sqrt[3]{27\beta_{1}\varepsilon^{2}{I_{0}}^{2}+18\Omega^{2}\mu+2\mu^{3}}}{3\sqrt[3]{2}\beta_{1}}\sqrt[3]{1+\sqrt{\frac{4\left( 3\Omega^{2}-\mu^{2} \right)^{3}}{\left( 27\beta_{1}\varepsilon^{2}{I_{0}}^{2}+18\Omega^{2}\mu+2\mu^{3} \right)^{2}}+1}}-\frac{\frac{\sqrt[3]{2}}{3}\left( 3\Omega^{2}-\mu^{2} \right)}{\sqrt[3]{27\beta_{1}^{4}\varepsilon^{2}{I_{0}}^{2}+18\beta_{1}^{3}\Omega^{2}\mu+2\beta_{1}^{3}\mu^{3}}}\frac{1}{\sqrt[3]{1+\sqrt{\frac{4\left( 3\Omega^{2}-\mu^{2} \right)^{3}}{\left( 27\beta_{1}\varepsilon^{2}{I_{0}}^{2}+18\Omega^{2}\mu+2\mu^{3} \right)^{2}}+1}}}-\frac{2\mu}{3\beta_{1}} \right)^{\frac{1}{2}} \left( A2.8 \right)$$

Assuming entrainment, the steady state phase offset of the main oscillator can be derived as follows:

$$\sin\psi_{ss}=\frac{Ωr_{ss}}{\varepsilon I_{0}}$$

$$\emptyset_{ss}-\omega_{0}t-\xi_{0}=\sin^{-1} \frac{Ωr_{ss}}{\varepsilon I_{0}}$$

$$\delta_{ss} \emptyset_{ss}-\omega_{0}t=\xi_{0}+\sin^{-1} \frac{Ωr_{ss}}{\varepsilon I_{0}}$$

**Appendix 3: Steady state dynamics of adaptive Hopf phase of first stage of training.**

The dynamics of a single CAO oscillator, in polar coordinates, can be approximated for the following condition ($A_{r}\ll\varepsilon I_{0}$):

$$\dot{r}\approx\left( \mu-\beta_{1}r^{2} \right)r+\varepsilon I_{0}\cos\psi=f_{1}(r, \psi,\omega) A3.1$$

$$\dot{\psi}\approx\Omega-\frac{\varepsilon I_{0}}{r}\sin\psi=f_{2}(r, \psi,\omega) A3.2$$

$$\dot{\omega}=-\eta_{\omega}\left( Re\left( I_{e}\left( t \right) \right)\sin\emptyset-Im\left( I_{e}\left( t \right) \right)\cos\emptyset\right)=f_{3}(r, \psi,\omega) A3.3$$

where, $I_{e}\left( t \right)=\varepsilon I\left( t \right)=\varepsilon I_{0}e^{i\left( \omega_{0}t+\xi_{0} \right)}$, the $\omega$ dynamics can be simplified as following:

$$\dot{\omega}=-\eta_{\omega}\left( Re\left( I_{e}\left( t \right) \right)\sin\emptyset-Im\left( I_{e}\left( t \right) \right)\cos\emptyset\right)$$

$$=-\eta_{\omega}\left( \varepsilon I_{0}\cos\left( \omega_{0}t+\xi_{0} \right)\sin\emptyset-\varepsilon I_{0}\sin\left( \omega_{0}t+\xi_{0} \right)\cos\emptyset\right)$$

$$\dot{\omega}=-\eta_{\omega}\varepsilon I_{0}\sin\psi$$

At steady state, $\dot{\omega_{ss}}=-\eta_{\omega}\varepsilon I_{0}\sin\psi_{ss}=0$. The stable solution of which would be, $\psi_{ss}^{*}=2n\pi$. Similarly, from eqn. A3.2,

$$\dot{\psi_{ss}}\approx\Omega_{ss}-\frac{\varepsilon I_{0}}{r_{ss}}\sin\psi_{ss}=0$$

$$i.e, \Omega_{ss}=0$$

$$or, \omega_{ss}=\omega_{0}$$

So, the steady state magnitude of oscillation will be the solution of the following expression as described in Appendix-2:

$$\left( \mu-\beta_{1}{r_{ss}}^{2} \right)r_{ss}+\varepsilon I_{0}\approx0$$

In this scenario the phase offset of the CAO oscillator at steady state will be:

$$\delta_{ss}=2\pi n+\xi_{0}$$

The corresponding Jacobian matrix for eqns. (A3.1-A3.3):

$$J=\left[ \begin{matrix} \frac{\partial f_{1}}{\partial r} & \frac{\partial f_{1}}{\partial\psi} & \frac{\partial f_{1}}{\partial\omega} \\ \frac{\partial f_{2}}{\partial r} & \frac{\partial f_{2}}{\partial\psi} & \frac{\partial f_{2}}{\partial\omega} \\ \frac{\partial f_{3}}{\partial r} & \frac{\partial f_{3}}{\partial\psi} & \frac{\partial f_{3}}{\partial\omega} \end{matrix} \right]$$

$$J=\left[ \begin{matrix} \mu-3\beta_{1}r^{2} & -\varepsilon I_{0}\sin\psi& 0 \\ \frac{\varepsilon I_{0}}{r^{2}}\sin\psi& -\frac{\varepsilon I_{0}}{r}\cos\psi& 1 \\ 0 & -\eta_{\omega}\varepsilon I_{0}\cos\psi& 0 \end{matrix} \right]$$

The Jacobian matrix at the fixed point or the steady state solution:

$$J=\left[ \begin{matrix} \mu-3\beta_{1}{r_{ss}^{*}}^{2} & 0 & 0 \\ 0 & -\frac{\varepsilon I_{0}}{r_{ss}^{*}} & 1 \\ 0 & -\eta_{\omega}\varepsilon I_{0} & 0 \end{matrix} \right]$$

Assuming $\beta_{1}<0$, the determinant ($\Delta$) and the trace ($T$) respectively:

$$\Delta=\eta_{\omega}\varepsilon I_{0}\left( \mu-3\beta_{1}{r_{ss}^{*}}^{2} \right)$$

$$T=\mu-3\beta_{1}{r_{ss}^{*}}^{2}-\frac{\varepsilon I_{0}}{r_{ss}^{*}}$$

So, the solution $r_{ss}^{*}$, $\omega_{ss}^{*}=\omega_{0}$ and $\psi_{ss}^{*}=2n\pi$ will be saddle point as $r_{ss}^{*}>\sqrt{\frac{\mu}{\beta_{1}}}$, which ensures $\mu-3\beta_{1}{r_{ss}^{*}}^{2}<0$, or $\Delta<0$.

**Appendix-4: Steady state dynamics of the transient phase of the second stage of training.**

The dynamics of the single unit of the network during the transient phase of the 2^nd^ stage of training is defined by eqns. 12, 13, 14. The complex variable counterpart of eqns. 12 and 13 are:

$$\dot{z}=z\left( \mu+i\omega-\beta_{1}\left| z \right|^{2} \right)+A_{r}e^{i\theta_{r}}{z_{r}}^{\frac{\omega^{*}}{\omega_{r}}}+\varepsilon I_{0}e^{i\left( \omega_{0}t+\xi_{0} \right)} A4.1$$

$$or, \dot{z}=z\left( \mu+i\omega-\beta_{1}\left| z \right|^{2} \right)+I_{r}\left( t \right)+I_{e}\left( t \right)$$

$$\dot{z_{r}}=z_{r}\left( \mu_{r}+i\omega_{r}-\beta_{1r}\left| z_{r} \right|^{2} \right) A4.2$$

The complex activation of the reference oscillator at steady state,

$$z_{rss}=r_{rss}e^{i\emptyset_{rss}}=\sqrt{\frac{\mu_{r}}{\beta_{1r}}}e^{i\left( \omega_{r}t+\emptyset_{r}\left( 0 \right) \right)}$$

Assuming the special condition, $\Omega<\Delta\omega$, $\omega\neq\omega_{0}$ and $\epsilon_{max}>\varepsilon I_{0}-A_{r}>\epsilon_{min}$ i.e., the CAO oscillator is operating under the entrainment regime of the input signal (where $\Delta\omega$ depends on the $\mu$, $\beta_{1}$, $\varepsilon$, $F$, $\xi_{0}$, $\theta_{r}$ and $A_{r}$) with a visible amount of interference between $I_{e}\left( t \right)$ and $I_{r}\left( t \right)$, at steady state, the frequency of the CAO oscillator becomes the frequency of the input signal i.e., $\dot{\emptyset}$ or $\omega^{*}$ becomes equal to $\omega_{0}$. So, the steady state version of eqn. A4.1 can be written as:

$$\dot{z}=z\left( \mu+i\omega-\beta_{1}\left| z \right|^{2} \right)+A_{r}e^{i\theta_{r}}{\frac{\mu_{r}}{\beta_{1r}}}^{\frac{\omega_{0}}{2\omega_{r}}}e^{i\left( \omega_{0}t+\emptyset_{r}\left( 0 \right)\frac{\omega_{0}}{\omega_{r}} \right)}+\varepsilon I_{0}e^{i\left( \omega_{0}t+\xi_{0} \right)}$$

$$=z\left( \mu+i\omega-\beta_{1}\left| z \right|^{2} \right)+A_{r}{\frac{\mu_{r}}{\beta_{1r}}}^{\frac{\omega_{0}}{2\omega_{r}}}e^{i\left( \omega_{0}t+\theta_{r}+\emptyset_{r}\left( 0 \right)\frac{\omega_{0}}{\omega_{r}} \right)}+\varepsilon I_{0}e^{i\left( \omega_{0}t+\xi_{0} \right)}$$

$$=z\left( \mu+i\omega-\beta_{1}\left| z \right|^{2} \right)+e^{i\omega_{0}t}\left( \varepsilon I_{0}e^{i\xi_{0}}+A_{r}{\frac{\mu_{r}}{\beta_{1r}}}^{\frac{\omega_{0}}{2\omega_{r}}}e^{i\left( \theta_{r}+\emptyset_{r}\left( 0 \right)\frac{\omega_{0}}{\omega_{r}} \right)} \right)$$

$$\dot{z}=z\left( \mu+i\omega-\beta_{1}\left| z \right|^{2} \right)+\bar{F}_{net}e^{i\omega_{0}t}$$

where,

$$\bar{F}_{net}=\left( \varepsilon I_{0}e^{i\xi_{0}}+A_{r}{\frac{\mu_{r}}{\beta_{1r}}}^{\frac{\omega_{0}}{2\omega_{r}}}e^{i\left( \theta_{r}+\emptyset_{r}\left( 0 \right)\frac{\omega_{0}}{\omega_{r}} \right)} \right)$$

is the phasor corresponding to the net input to the CAO oscillator at steady-state. The magnitude and the phase offset of $\bar{F}_{net}$ are:

$$\left| \bar{F}_{net} \right|=\left( \left( \varepsilon I_{0}\cos\xi_{0}+A_{r}{\frac{\mu_{r}}{\beta_{1r}}}^{\frac{\omega_{0}}{2\omega_{r}}}\cos\left( \theta_{r}+\emptyset_{r}\left( 0 \right)\frac{\omega_{0}}{\omega_{r}} \right) \right)^{2}+\left( \varepsilon I_{0}\sin\xi_{0}+A_{r}{\frac{\mu_{r}}{\beta_{1r}}}^{\frac{\omega_{0}}{2\omega_{r}}}\sin\left( \theta_{r}+\emptyset_{r}\left( 0 \right)\frac{\omega_{0}}{\omega_{r}} \right) \right)^{2} \right)^{0.5} \left( A4.3 \right)$$

$$arg\left( \bar{F}_{net} \right)=\tan^{-1} \frac{\varepsilon I_{0}\sin\xi_{0}+A_{r}{\frac{\mu_{r}}{\beta_{1r}}}^{\frac{\omega_{0}}{2\omega_{r}}}\sin\left( \theta_{r}+\emptyset_{r}\left( 0 \right)\frac{\omega_{0}}{\omega_{r}} \right)}{\varepsilon I_{0}\cos\xi_{0}+A_{r}{\frac{\mu_{r}}{\beta_{1r}}}^{\frac{\omega_{0}}{2\omega_{r}}}\cos\left( \theta_{r}+\emptyset_{r}\left( 0 \right)\frac{\omega_{0}}{\omega_{r}} \right)} \left( A4.4 \right)$$

The steady-state magnitude of oscillation is dependent on $\left| \bar{F}_{net} \right|$ whereas the phase offset of oscillation $\delta_{ss}=$ ($\emptyset-\omega^{*}t$) will be same as $arg\left( \bar{F}_{net} \right)$ which is verified in fig. 10C.

**Appendix-5: Steady state dynamics of Hebbian plasticity phase of second stage of training.**

The approximate dynamics of the single unit at Hebbian plasticity phase of the 2^nd^ stage with the assumption $\varepsilon I_{0}\gg A_{r}$:

$$\dot{r}\approx r\left( \mu-\beta_{1}r^{2} \right)+\varepsilon I_{0}\cos\psi=f_{1} A5.1$$

$$\dot{\psi}\approx\Omega-\frac{\varepsilon I_{0}}{r}\sin\psi=f_{2} A5.2$$

$$\tau_{\omega}\dot{\omega^{*}}\approx-\omega^{*}+\omega-\frac{\varepsilon I_{0}}{r}\sin\psi A5.3$$

$$\dot{r_{r}}=\left( \mu_{r}-\beta_{1r}{r_{r}}^{2} \right)r_{r} A5.4$$

$$\dot{\emptyset_{r}}=\omega_{r} A5.5$$

$$\dot{\theta_{r}}=-\frac{r{r_{r}}^{\frac{\omega^{*}}{\omega_{r}}}}{\tau_{W}A_{r}}\sin\omega^{*}\left( \frac{\theta_{r}}{\omega^{*}}+\frac{\emptyset_{r}}{\omega_{r}}-\frac{\emptyset}{\omega^{*}} \right)=f_{3} A5.6$$

Considering the CAO oscillator is operating inside the entrainment regime, at steady state $\omega^{*}$ becomes $\omega_{0}$. From equation-$A5.2$ we get;

$$\sin\psi_{ss}=\frac{\Omega r_{ss}}{\varepsilon I_{0}}$$

So, $\psi_{ss}^{*}=\sin^{-1} \left( \frac{\Omega r_{ss}}{\varepsilon I_{0}} \right)$, $\cos\psi_{ss}=\sqrt{1-\left( \frac{\Omega r_{ss}}{\varepsilon I_{0}} \right)^{2}}$ and the phase offset of the CAO oscillator at steady-state $\delta_{ss}^{*}=\xi_{0}+\sin^{-1} \left( \frac{\Omega r_{ss}}{\varepsilon I_{0}} \right)$. The steady state dynamics of equation $A5.6$:

$$\dot{\theta_{r}}=-\frac{r{r_{r}}^{\frac{\omega^{*}}{\omega_{r}}}}{\tau_{W}A_{r}}\sin\omega^{*}\left( \frac{\theta_{r}}{\omega^{*}}+\frac{\emptyset_{r}}{\omega_{r}}-\frac{\emptyset}{\omega^{*}} \right)=0$$

$$\frac{r_{ss}{r_{rss}}^{\frac{\omega_{0}}{\omega_{r}}}}{\tau_{W}A_{r}}\sin\omega_{0}\left( \frac{\theta_{rss}}{\omega_{0}}+\frac{\emptyset_{rss}}{\omega_{r}}-\frac{\emptyset_{ss}}{\omega_{0}} \right)=0$$

$$\sin\omega_{0}\left( \frac{\theta_{rss}}{\omega_{0}}+\frac{\emptyset_{rss}}{\omega_{r}}-\frac{\emptyset_{ss}}{\omega_{0}} \right)=0$$

$$\omega_{0}\left( \frac{\theta_{rss}}{\omega_{0}}+\frac{\emptyset_{rss}}{\omega_{r}}-\frac{\emptyset_{ss}}{\omega_{0}} \right)=n\pi A5.8$$

At steady state, $\psi_{ss}=\sin^{-1} \left( \frac{\Omega r_{ss}}{\varepsilon I_{0}} \right)$ or, $\emptyset_{ss}=\omega_{0}t+\xi_{0}+\sin^{-1} \left( \frac{\Omega r_{ss}}{\varepsilon I_{0}} \right)$, and $\emptyset_{rss}=\omega_{r}t$, assuming $\emptyset_{r}\left( 0 \right)=0$, substituting the values of $\emptyset_{rss}$ and $\emptyset_{ss}$ into equation A4.8.

$$\theta_{rss}+\omega_{0}t-\omega_{0}t-\xi_{0}-\sin^{-1} \left( \frac{\Omega r_{ss}}{\varepsilon I_{0}} \right)=n\pi$$

$$\theta_{rss}=n\pi+\xi_{0}+\sin^{-1} \left( \frac{\Omega r_{ss}}{\varepsilon I_{0}} \right) A5.9$$

Likewise, $r_{ss}^{*}$ would be the solution of the following expression:

$$\left( \mu+\beta_{1}{r_{ss}}^{2} \right)r_{ss}+\varepsilon F\sqrt{1-\left( \frac{\Omega r_{ss}}{\varepsilon I_{0}} \right)^{2}}=0$$

$${\beta_{1}}^{2}{r_{ss}}^{6}+2\mu\beta_{1}{r_{ss}}^{4}+\left( \mu^{2}+Ω^{2} \right){r_{ss}}^{2}-\varepsilon^{2}{I_{0}}^{2}=0$$

**Appendix-6: Steady state dynamics of Hebbian plasticity phase of 2^nd^ stage of training under the condition** $A_{r}\neq0$:

Polar coordinate representation of the dynamics:

$$\dot{r}=\left( \mu-\beta_{1}r^{2} \right)r+A_{r}{r_{r}}^{\frac{\omega^{*}}{\omega_{r}}}\cos\omega^{*}\left( \frac{\emptyset}{\omega^{*}}-\frac{\theta_{r}}{\omega^{*}}-\frac{\emptyset_{r}}{\omega_{r}} \right)+\varepsilon I_{0}\cos\left( \omega_{0}t+\xi_{0}-\emptyset\right) (A6.1)$$

$$\dot{\emptyset}=\omega-A_{r}\frac{{r_{r}}^{\frac{\omega^{*}}{\omega_{r}}}}{r}\sin\omega^{*}\left( \frac{\emptyset}{\omega^{*}}-\frac{\theta_{r}}{\omega^{*}}-\frac{\emptyset_{r}}{\omega_{r}} \right)+\frac{\varepsilon I_{0}}{r}\sin\left( \omega_{0}t+\xi_{0}-\emptyset\right) (A6.2)$$

$$\dot{r_{r}}=r_{r}\left( \mu_{r}-\beta_{1r}{r_{r}}^{2} \right) (A6.3)$$

$$\dot{\emptyset_{r}}=\omega_{r} (A6.4)$$

$$\tau_{\omega}\dot{\omega^{*}}=-\omega^{*}+\omega-A_{r}\frac{{r_{r}}^{\frac{\omega^{*}}{\omega_{r}}}}{r}\sin\omega^{*}\left( \frac{\emptyset}{\omega^{*}}-\frac{\theta_{r}}{\omega^{*}}-\frac{\emptyset_{r}}{\omega_{r}} \right)+\frac{\varepsilon I_{0}}{r}\sin\left( \omega_{0}t+\xi_{0}-\emptyset\right) (A6.5)$$

$$\tau_{W}\dot{\theta_{r}}=\frac{r{r_{r}}^{\frac{\omega^{*}}{\omega_{r}}}}{A_{r}}\sin\omega^{*}\left( \frac{\emptyset}{\omega^{*}}-\frac{\theta_{r}}{\omega^{*}}-\frac{\emptyset_{r}}{\omega_{r}} \right) (A6.6)$$

A brief analysis of the steady state dynamics as follows:

Considering the CAO oscillator is operating inside the entrainment regime, at steady state $\omega^{*}$ becomes $\omega_{0}$. Writing eqns. A6.1 and A6.2 in terms of $\psi$ and $\Omega$.

$$\dot{r}=\left( \mu-\beta_{1}r^{2} \right)r+A_{r}{r_{r}}^{\frac{\omega^{*}}{\omega_{r}}}\cos\omega^{*}\left( \frac{\emptyset}{\omega^{*}}-\frac{\theta_{r}}{\omega^{*}}-\frac{\emptyset_{r}}{\omega_{r}} \right)+\varepsilon I_{0}\cos\psi$$

$$\dot{\psi}=\Omega-\frac{{r_{r}}^{\frac{\omega_{*}}{\omega_{r}}}}{r}\sin\omega^{*}\left( \frac{\emptyset}{\omega^{*}}-\frac{\theta_{r}}{\omega^{*}}-\frac{\emptyset_{r}}{\omega_{r}} \right)-\frac{\varepsilon I_{0}}{r}\sin\psi$$

In the entrainment regime, $\omega^{*}$ becomes $\omega_{0}$ at steady state. From eqn. A6.6 at steady state:

$$\frac{r_{ss}{r_{rss}}^{\frac{\omega_{0}}{\omega_{r}}}}{A_{r}}\sin\omega_{0}\left( \frac{\emptyset}{\omega_{0}}-\frac{\theta_{rss}}{\omega_{0}}-\frac{\emptyset_{r}}{\omega_{r}} \right)=0$$

$\sin\omega_{0}\left( \frac{\emptyset}{\omega_{0}}-\frac{\theta_{rss}}{\omega_{0}}-\frac{\emptyset_{r}}{\omega_{r}} \right)=0$ or, $\cos\omega_{0}\left( \frac{\emptyset}{\omega_{0}}-\frac{\theta_{rss}}{\omega_{0}}-\frac{\emptyset_{r}}{\omega_{r}} \right)=1$

$$\frac{\boldsymbol{\theta}_{\boldsymbol{rss}}}{\boldsymbol{\omega}_{\boldsymbol{0}}}\boldsymbol{=}\frac{\boldsymbol{\emptyset}}{\boldsymbol{\omega}_{\boldsymbol{0}}}\boldsymbol{-}\frac{\boldsymbol{\emptyset}_{\boldsymbol{r}}}{\boldsymbol{\omega}_{\boldsymbol{r}}}\boldsymbol{(A}\boldsymbol{6.8)}$$

From eqn. A6.2 at steady state;

$$\Omega-\frac{{r_{rss}}^{\frac{\omega_{0}}{\omega_{r}}}}{r_{ss}}\sin\omega_{0}\left( \frac{\emptyset}{\omega_{0}}-\frac{\theta_{rss}}{\omega_{0}}-\frac{\emptyset_{r}}{\omega_{r}} \right)-\frac{\varepsilon I_{0}}{r_{ss}}\sin\psi_{ss}=0$$

$$\psi_{ss}=\sin^{-1} \frac{\Omega r_{ss}}{\varepsilon I_{0}}$$

$$\emptyset_{ss}=\omega_{0}t+\xi_{0}+\sin^{-1} \frac{\Omega r_{ss}}{\varepsilon I_{0}} (A6.9)$$

From eqns. A6.8 and A6.9 assuming the reference oscillator was initialized at 0 phase, $\emptyset_{r}\left( 0 \right)=0$.

$$\frac{\theta_{rss}}{\omega_{0}}=\frac{\xi_{0}}{\omega_{0}}+\frac{1}{\omega_{0}}\sin^{-1} \frac{\Omega r_{ss}}{\varepsilon I_{0}}$$

$$\boldsymbol{\theta}_{\boldsymbol{rss}}\boldsymbol{=}\boldsymbol{\xi}_{\boldsymbol{0}}\boldsymbol{+}\mathbf{sin}^{\boldsymbol{-1}} \frac{\boldsymbol{\Omega}\boldsymbol{r}_{\boldsymbol{ss}}}{\boldsymbol{\varepsilon}I_{0}}$$

From eqn. A6.1 we can find the steady state value of $r$ by finding the solution of the following equation;

$$\left( \mu+\beta_{1}{r_{ss}}^{2} \right)r_{ss}+A_{r}{r_{rss}}^{\frac{\omega_{0}}{\omega_{r}}}+\varepsilon I_{0}\sqrt{1-\left( \frac{\Omega r_{ss}}{\varepsilon I_{0}} \right)^{2}}=0$$

# Figures


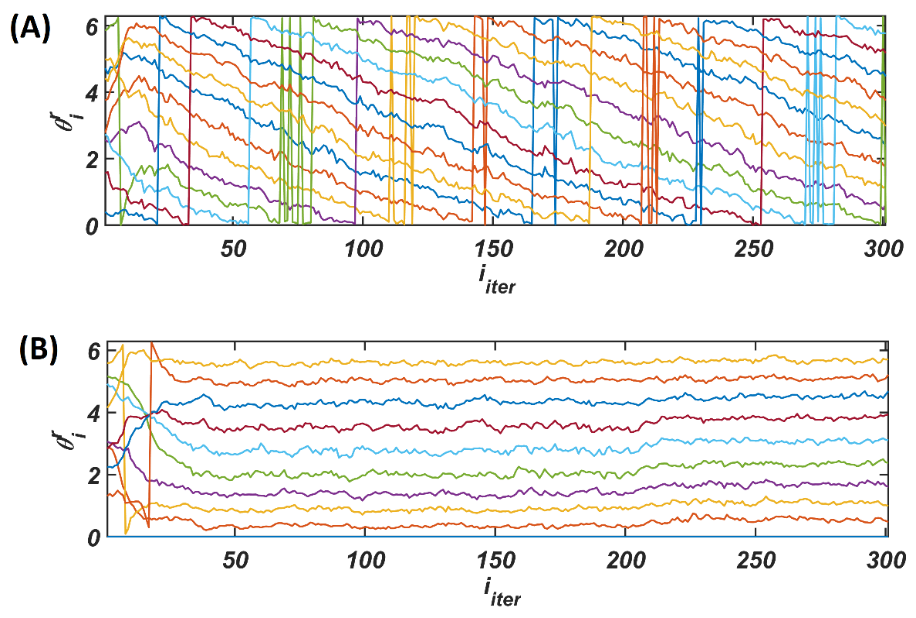


**Figure S1:** **(A)** Evolution of the $\theta_{ij}^{r}$s of a given column over multiple epochs in the 2^nd^ stage of training with the periodic CAO along the dimension of y-axis, **(B)** Self organization of $\theta_{ij}^{r}$s of the same column throughout the 2^nd^ stage of training with the periodic CAO along the dimension of y-axis along with fixed $\theta_{ij}^{r}$s of the top most row with $0^{o}$ angle.

# Tables

| Variable Notations and Abbreviations | Variable Representations |
| --- | --- |
| SRO | Subcortical Reference Oscillator |
| CAO | Cortical Array of Oscillators |
| $z_{ij}$ | Complex activation of the oscillator located in $i^{th}$ row and $j^{th}$ column in the CAO |
| $x_{ij},y_{ij}$ | Real and imaginary part of $z_{ij}$ respectively |
| $r_{ij},\emptyset_{ij}$ | Magnitude and angle of $z_{ij}$ respectively |
| $z_{r}$ | Complex activation of the SRO |
| $x_{r},y_{r}$ | Real and imaginary part of $z_{r}$ respectively |
| $r_{r},\emptyset_{r}$ | Magnitude and angle of $z_{r}$ respectively |
| $I$ | External input to the oscillators in CAO |
| $I_{0},\emptyset_{0},\omega_{0},\xi_{0}$ | Magnitude, phase, frequency and phase offset of $I$ respectively |
| $I_{e},I_{ij}^{r} or I_{r}$ | Overall external input and overall input from the reference oscillator to the oscillator located in $i^{th}$ row and $j^{th}$ column in the CAO respectively |
| $a_{r}, a_{0}$ | Magnitudes of $I_{r}$ and $I_{e}$ respectively |
| $\xi_{0},\xi_{r}$ | Phase offsets of $I_{e}$ and $I_{r}$ respectively |
| $I_{ij}^{t} or I_{t}$ | Overall input to the CAO oscillator |
| $\bar{F}_{net} or \bar{F}_{ij}^{net}$ | Resultant input to the oscillator at $(i,j)$ in CAO under the 1^st^ condition, i.e., $I_{ij}^{t}=e^{i\omega_{ij}t}\bar{F}_{ij}^{net}$ |
| $\psi,\Omega$ | Relative phase and the frequency of the CAO oscillator w.r.t the input respectively |
| $\lambda_{r}$ | Normalized phase difference between the CAO oscillator and the SRO, |
| $N_{x}$ | No of columns in the OTSOM model |
| $N_{y}$ | No of rows in the OTSOM model |
| $\varepsilon$ | Magnitude of the uniform real afferent weights |
| $W_{ij}^{r}$ | Complex unilateral modified coupling coefficient from the SRO to the oscillator located in $i^{th}$ row and $j^{th}$ column in the CAO |
| $A_{ij}^{r},\theta_{ij}^{r}$ | Magnitude and angle of $W_{ij}^{r}$ respectively |
| $\mu_{r},\beta_{1r},\omega_{r}$ | Intrinsic dynamical parameters of the SRO |
| $\mu,\beta_{1},\omega_{ij}$ | Intrinsic dynamical parameters of the oscillator located in $i^{th}$ row and $j^{th}$ column in the CAO |
| $Ʊ_{\omega}$ | Set of the sampled frequencies of external input signals |
| $Ʊ_{\xi}$ | Set of the sampled phase offset of the external input signal |
| $N_{\omega}$ | No of sampled frequencies in the set $Ʊ_{\omega}$ |
| $N_{\xi}$ | No of sampled phase offsets in the set $Ʊ_{\xi}$ |
| $N$ | Total no of external input signals with all combinations of frequencies and phase offsets from the sets $Ʊ_{\omega}$ and $Ʊ_{\xi}$ respectively. |
| $\omega_{0p}, \xi_{0p}$ | Frequency and the phase offset of the $p^{th}$ pattern sampled from $Ʊ_{\omega}$ and $Ʊ_{\xi}$ respectively |
| $N_{epoch,\omega},N_{epoch,\xi}$ | No of training epochs during the 1^st^ stage and 2^nd^ stage of training respectively |
| $i_{epoch}$ | Index of the training epoch |
| $T_{s\omega}$ | Duration of the Transient phase of the 1^st^ stage of training |
| $T_{s\theta}$ | Duration of the Transient phase of the 2^nd^ stage of training |
| $T_{t\omega}$ | Duration of the Adaptive-Hopf phase of the 1^st^ stage of training |
| $T_{h\theta}$ | Duration of the Hebbian Plasticity phase of the 2^nd^ stage of training |
| ${\eta_{\omega}}_{ij}^{mn},{\eta_{\theta}}_{ij}^{mn}$ | Gaussian neighbourhood function during the 1^st^ and 2^nd^ stages of training respectively |
| $W_{ij}^{mn}$ | Neighbourhood window |
| $\sigma_{y\omega},\sigma_{x\omega}$ | Standard deviations along the rows and columns respectively of ${\eta_{\omega}}_{ij}^{mn}$. |
| $\sigma_{y\theta},\sigma_{x\theta}$ | Standard deviations along the rows and columns respectively of ${\eta_{\theta}}_{ij}^{mn}$ |
| ${\eta_{\omega}}_{0}$ | Frequency learning rate of the winner oscillator during the 1^st^ phase of training |
| ${\eta_{\theta}}_{max}$, ${\eta_{\theta}}_{min}$ | Maximum and the minimum values of phase learning rate of the winner oscillator during the 2^nd^ phase of training |
| $\sigma_{y\omega m},\sigma_{y\theta m}$ | Maximum values of $\sigma_{y\omega}$ and $\sigma_{y\theta}$ respectively |
| $\sigma_{x\omega m},\sigma_{x\theta m}$ | Maximum values of $\sigma_{x\omega}$ and $\sigma_{x\theta}$ respectively |
| $\sigma_{\sigma x\omega},\sigma_{\sigma y\omega}$ | Standard deviations of $\sigma_{x\omega}$ and $\sigma_{y\omega}$ respectively w.r.t the iteration index |
| $\sigma_{\sigma x\theta},\sigma_{\sigma y\theta}$ | Standard deviations of $\sigma_{x\theta}$ and $\sigma_{y\theta}$ respectively w.r.t the iteration index |
| $d_{r\omega},d_{c\omega}$ | Half-lengths of the neighbourhood window along rows and columns respectively during the 1^st^ stage of training |
| $d_{r\theta},d_{c\theta}$ | Half-lengths of the neighbourhood window along rows and columns respectively during the 2^nd^ stage of training |
| $\tau_{\omega}$ | Time constant for $\omega^{*}$ |
| $\omega^{*}$ | Actual frequency of oscillation of the oscillator |

**Table-S1:** The table lists down all the variables, parameters and the abbreviations, the values they are representing and the corresponding notations.
